# DNA Methylation, Deamination, and Translesion Synthesis Combine to Generate Footprint Mutations in Cancer Driver Genes in B-Cell Derived Lymphomas and Other Cancers

**DOI:** 10.3389/fgene.2021.671866

**Published:** 2021-05-19

**Authors:** Igor B. Rogozin, Abiel Roche-Lima, Kathrin Tyryshkin, Kelvin Carrasquillo-Carrión, Artem G. Lada, Lennard Y. Poliakov, Elena Schwartz, Andreu Saura, Vyacheslav Yurchenko, David N. Cooper, Anna R. Panchenko, Youri I. Pavlov

**Affiliations:** ^1^National Center for Biotechnology Information, National Library of Medicine, National Institutes of Health, Bethesda, MD, United States; ^2^Center for Collaborative Research in Health Disparities – RCMI Program, University of Puerto Rico, San Juan, Puerto Rico; ^3^Department of Pathology and Molecular Medicine, School of Medicine, Queen’s University, Kingston, ON, Canada; ^4^Integrated Informatics Services Core – RCMI, University of Puerto Rico, San Juan, Puerto Rico; ^5^Department Microbiology and Molecular Genetics, University of California, Davis, Davis, CA, United States; ^6^Life Science Research Centre, Faculty of Science, University of Ostrava, Ostrava, Czechia; ^7^Coordinating Center for Clinical Trials, National Cancer Institute, National Institutes of Health, Bethesda, MD, United States; ^8^Martsinovsky Institute of Medical Parasitology, Tropical and Vector Borne Diseases, Sechenov First Moscow State Medical University, Moscow, Russia; ^9^Institute of Medical Genetics, Cardiff University, Cardiff, United Kingdom; ^10^Eppley Institute for Research in Cancer and Allied Diseases, Omaha, NE, United States; ^11^Department of Microbiology and Pathology, Biochemistry and Molecular Biology, Genetics, Cell Biology and Anatomy, University of Nebraska Medical Center, Omaha, NE, United States; ^12^ Department of Genetics and Biotechnology, Saint-Petersburg State University, Saint-Petersburg, Russia

**Keywords:** tumor cells, frequency matrices, database, computational biology, somatic hypermutation, immunoglobulin genes, gene expression

## Abstract

Cancer genomes harbor numerous genomic alterations and many cancers accumulate thousands of nucleotide sequence variations. A prominent fraction of these mutations arises as a consequence of the off-target activity of DNA/RNA editing cytosine deaminases followed by the replication/repair of edited sites by DNA polymerases (pol), as deduced from the analysis of the DNA sequence context of mutations in different tumor tissues. We have used the weight matrix (sequence profile) approach to analyze mutagenesis due to Activation Induced Deaminase (AID) and two error-prone DNA polymerases. Control experiments using shuffled weight matrices and somatic mutations in immunoglobulin genes confirmed the power of this method. Analysis of somatic mutations in various cancers suggested that AID and DNA polymerases η and θ contribute to mutagenesis in contexts that almost universally correlate with the context of mutations in A:T and G:C sites during the affinity maturation of immunoglobulin genes. Previously, we demonstrated that AID contributes to mutagenesis in (de)methylated genomic DNA in various cancers. Our current analysis of methylation data from malignant lymphomas suggests that driver genes are subject to different (de)methylation processes than non-driver genes and, in addition to AID, the activity of pols η and θ contributes to the establishment of methylation-dependent mutation profiles. This may reflect the functional importance of interplay between mutagenesis in cancer and (de)methylation processes in different groups of genes. The resulting changes in CpG methylation levels and chromatin modifications are likely to cause changes in the expression levels of driver genes that may affect cancer initiation and/or progression.

## Introduction

Epigenetic reprogramming in cancer genomes creates a distinct DNA methylation landscape encompassing clustered sites of hypermethylation at regulatory regions and protein-coding genes separated by long intergenic tracks of hypomethylated regions. Such changes in DNA methylation landscape are displayed by most cancer types, and hence have the potential to serve as a universal cancer biomarker (Sina et al., 2018; Oliver et al., 2021). Previous research has focused on the biological consequences of DNA methylation changes in genomes, whereas its impact on the structure and flexibility of DNA, and its vulnerability to modifications/repair/replication in cancer, have remained largely unexplored.

Other prominent features of cancer initiation and progression are genomic alterations. Cancer genomes harbor numerous genomic alterations, including hundreds/thousands of nucleotide sequence variations (Stratton et al., 2009; Roberts and Gordenin, 2014; Rogozin et al., 2018c). A prominent fraction of these mutations arises as a consequence of the off-target activity of enzymes participating in somatic hypermutation (SHM) in immunoglobulin (Ig) genes: DNA/RNA editing cytosine deaminases of the Activation Induced Deaminase (AID)/APOBEC family and the replication/repair of edited sites by DNA polymerases (pols), as deduced by the analysis of the DNA sequence context of mutations in different cancer tissues (Alexandrov et al., 2013; Roberts and Gordenin, 2014; Swanton et al., 2015; Granadillo Rodriguez et al., 2020). Analyses of various types of cancer by means of this technique has yielded a set of 30–50 distinct mutation signatures implying many mechanisms of hypermutation in cancer cells (Alexandrov and Stratton, 2014; Goncearenco et al., 2017; Rogozin et al., 2018c; Islam and Alexandrov, 2021).

There is a well-established association between DNA methylation and genomic alteration. Early studies revealed that methylated cytosines explain mutation hotspots in bacteria (Coulondre et al., 1978). In eukaryotic genomes, CpG sites are known to be vulnerable to mutation in both cancer and normal cells (Cooper and Youssoufian, 1988; Alsøe et al., 2017; Goncearenco et al., 2017; Rogozin et al., 2018c; Brinkman et al., 2019). We recently detected a substantial excess of mutations in CpG sites that overlap with AID mutable motifs (WRC/GYW, W = A or T, R = A or G, Y = T or C, the mutable position is underlined) forming “hybrid” mutable motifs (WRCG/CGYW) whereas the opposite trend was observed in SHM (Rogozin and Diaz, 2004; Rogozin et al., 2016). This finding implies that in many cancers the SHM machinery acts aberrantly at genomic sites containing methylated cytosine. The discovery of abundant mutations in WRCG/CGYW motifs in many types of human cancer suggests that AID-mediated, CpG methylation-dependent mutagenesis is a common feature of tumorigenesis connecting methylation and hypermutation (Rogozin et al., 2016).

A prominent feature of carcinogenesis is the presence of cancer driver and passenger mutations. A driver mutation directly or indirectly confers a selective advantage upon cancer cells, whilst a passenger mutation does not (Stratton et al., 2009). In this context, it should be appreciated that there is a difference between a driver gene and a driver gene mutation: a driver gene may accumulate recurrent driver mutations but may also harbor passenger mutations. Some genes contain only recurrent passenger mutations with frequencies comparable to driver genes (hotspots related to the intrinsic properties of the processes of mutagenesis), which complicates the identification of cancer driver mutations (Rogozin et al., 2018c). In this study, we operationally define a non-driver gene as a gene that contains numerous mutations that do not cause cancer and are classified as passenger mutations according to the MutaGene (Goncearenco et al., 2017; Brown et al., 2019) and CHASMplus (Tokheim and Karchin, 2019) computational tools.

We studied the association of mutable motifs produced by AID and two error-prone DNA pols ultimately associated with cancer, and the methylation status of sets of driver and non-driver genes. Our null hypothesis was that driver and non-driver genes would have contrasting methylation and mutation profiles, which could be studied using mutable motifs (Rogozin et al., 2016). The conventional method for the analysis of mutable DNA motifs is the consensus approach (Alexandrov and Stratton, 2014), for example, 5′WRC for the AID enzyme (Pham et al., 2011; Rogozin et al., 2018c) or 5′WA for DNA pol η (Rogozin et al., 2001, 2018b). Here, we applied the weight matrix (sequence profile) approach that is frequently used in the analysis of biological processes (Rogozin et al., 2019) to the analysis of methylation profiles and mutagenesis generated by AID and error-prone DNA pols η and θ in CpG dinucleotides. Control experiments, using shuffled sites and SHM in immunoglobulin genes, suggested that the weight matrix method adds power to the study of mutagenesis. Analysis of mutations in various cancers indicated that AID and DNA pol η mutable motifs almost universally correlate with SHM in G:C sites. Analysis of mutations and motifs in A:T sites yielded a similar correlation for pol θ. Analysis of methylation data in malignant lymphomas (the MALY-DE dataset) suggested that the methylation status of driver genes differs from that of non-driver genes and this may be one reason for the differences in distribution of mutations in the two groups of genes.

## Materials and Methods

### Mutable Motif Construction Using Weight Matrices

**FIGURE 1:**
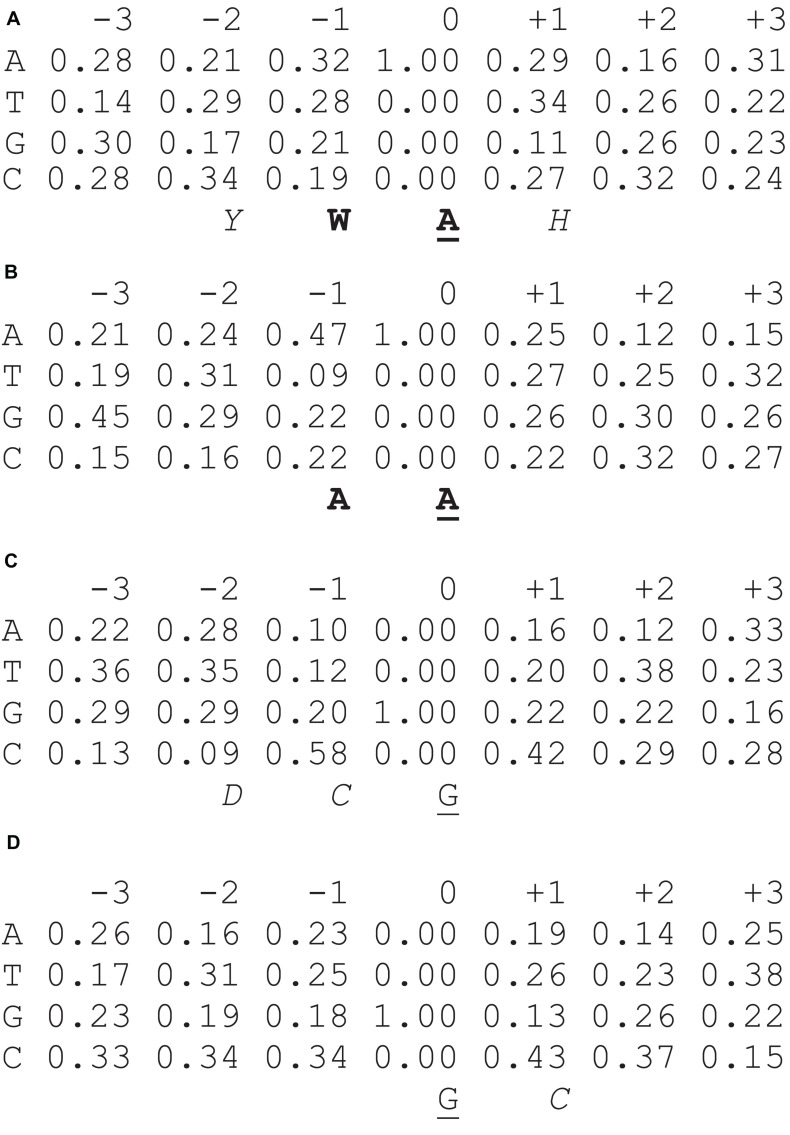
Nucleotide frequency matrices for mutations at A:T sites [**(A)** DNA pol η; **(B)** pol θ] and G:C sites [**(C)** pol θ; **(D)** DNA pol η]. Known mutable motifs (consensus sequences) (Matsuda et al., 2001; Rogozin et al., 2001) are shown below each matrix in bold, whereas mutable positions are underlined. Putative (previously unobserved) parts of mutable motifs and potentially informative positions are italicized, W = A or T; Y = T or C; B = A, T or G; D = A, T, or G. Source of data: Supplementary Figures 2, 3.

Several approaches have been developed for the analysis of a set of mutated genomic sequences (Staden, 1984; Rogozin et al., 2018b, 2019). A mononucleotide weight matrix is a simple and straightforward way to present the structure of a functional signal and to calculate weights for the signal sequence (Gelfand, 1995). Each matrix W(b,j) (nucleotide b = A, T, G, or C in a position j) includes information on a normalized frequency of A, T, G, and C bases in each of the six positions surrounding detected sites of mutation (3 bases downstream and 3 bases upstream; Figure 1; corresponding raw numbers are shown in the Supplementary Figure 1). We calculated the weight matrices for the two studied DNA polymerases and used a collection of mutations generated by classic gap-filling DNA synthesis *in vitro* by human pols η and θ (Matsuda et al., 2001; Rogozin et al., 2001; Arana et al., 2008) (Supplementary Figures 2, 3).

The following formula for W(b,j) was used for data analysis: W(b,j) = log_2_ [f(b,j) / e(b)], where f(b,j) is the observed frequency of the nucleotide b in position j and e(b) is the expected frequency of the nucleotide b calculated as the mean nucleotide frequencies of positions –5,–4, +4, +5 for the sites of mutation in the target sequence; the resulting W(b,j) matrices are shown in Figure 1.

The matching score S_(b1,…,bL)_ of a sequence b1,…,bL is:

$${\text{S}}_{\left(\text{b}\,1,\ldots ,\text{bL}\right)}=\sum_{\text{j}=1}^{\text{L}}\text{W}\,\left(\text{b},\text{j}\right)$$

The matching score between sequence b1,…,bL and a weight matrix can be further expressed as a percentage:

$$\%\mathrm{matching}\mathrm{score}=100\times \left({\text{S}}_{\left(\text{b}\,1,\ldots ,\text{bL}\right)}-{\text{S}}_{\min}\right)/\left({\text{S}}_{\max}-{\text{S}}_{\min}\right)$$

$${\text{S}}_{\left(\min\right)}=\sum_{\text{j}=1}^{\text{L}}\underset{\text{b}}{\text{MIN}}\left(\text{W}\,\left(\text{b},j\right)\right)$$

$${\text{S}}_{\left(\max\right)}=\sum_{\text{j}=1}^{\text{L}}\underset{\text{b}}{\text{MAX}}\left(\text{W}\,\left(\text{b},j\right)\right)$$

Hereafter, we use the term “weight” instead of “% matching score.” We used positions –3 : +3 to estimate the weights of sites.

### ICGC/TCGA Mutation Datasets

Somatic mutation data from the ICGC and TCGA cancer genome projects were extracted from the Sanger COSMIC Whole Genome Project v75.^1^ The ICGC/TCGA datasets are almost exclusively passenger mutations and, as such, they are unlikely to be subject to selection in the context of promoting cellular proliferation. Indeed, they are much more likely to reflect unselected mutational spectra (Goncearenco et al., 2017; Rogozin et al., 2018c). The tissues and cancer types were defined according to the primary tumor site and the cancer project in question. This dataset is included in the MutaGene package, where it is described in detail (Goncearenco et al., 2017; Brown et al., 2019). We also used collections of mutations obtained by means of *in vitro* experiments for human pol η (Matsuda et al., 2001) and pol θ (Arana et al., 2008; Supplementary Figures 2, 3) to build weight matrices.

### Methylation and Expression Data

**FIGURE 2:**
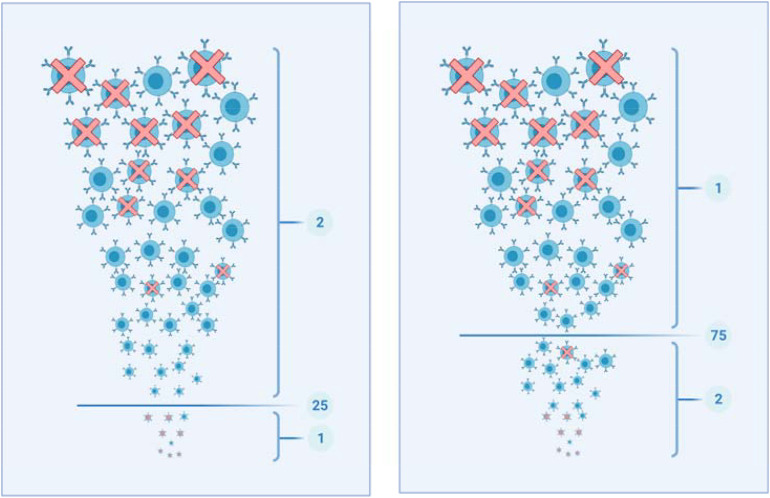
Schematic representation of the procedure used for construction of Table 3. Each circle represents a methylated CpG site, with its size reflecting the methylation level. Red “X” denotes CpG sites that overlap with known mutable motifs. The left and right panels correspond to thresholds 25% and 75%. The left panel: The set “1” (the methylation levels are smaller than 25%) is compared to set “2” (the methylation levels are larger than 25%). The right panel: The set “1” (the methylation levels are larger than 75%) is compared to set “2” (the methylation levels are smaller than 75%).

For the analysis of the association between somatic mutations, mRNA expression, mutable motifs and methylation, datasets for 26 patients with malignant lymphoma^2^ were used. In the analyzed datasets, the methylation data for all patients were pooled together. Each position was characterized by the methylated/unmethylated read count and the methylation ratio (the number of methylated reads divided by the total number of reads overlapping this position and multiplied by 100). Only positions with more than nine associated reads were included in the analysis. The major methodological problem inherent in the analysis of methylation across CpG’s is the absence of control sets. Therefore, we compared methylation values below and above threshold values (25 and 75%). The mean weight of mutable motifs (Figure 1) in the positions of methylated CpG’s (the group 1 with the size S1, Figure 2) was compared to the mean weight of the same motifs in a contrasting dataset (the group 2 with the size S2, Figure 2) using the *t*-test (2-tailed test) and Monte Carlo test (MC, 1-tailed test) similar to the consensus method as previously described (Rogozin et al., 2018b). Expression of mRNA was measured using the FPKM values (Howe et al., 2011). The mean and variance for each gene were calculated across 26 studied samples.

### Analysis of Mutations

DNA sequences surrounding the mutated nucleotides represent the mutation context. We compared the frequency of known mutable motifs for somatic mutations with the frequency of these motifs in the vicinity of the mutated nucleotide. Specifically, for each base substitution, the 121 bp sequence centered at the mutation was extracted (the DNA neighborhood). We used only the nucleotides immediately flanking the mutations because DNA repair/replication enzymes are thought to scan a very limited region of DNA (Roberts et al., 2013; Goncearenco et al., 2017; Rogozin et al., 2018c). This approach does not exclude any specific area of the genome, but rather uses the areas within each sample where mutagenesis has occurred (considering the variability in the mutation rate across the human genome) (Roberts et al., 2013; Rogozin et al., 2018b). A schematic representation of this procedure for CpG dinucleotides is shown in Supplementary Figure 4). Here, the mean weight of mutable motifs (represented by weight matrices; Figure 1) in the positions of each somatic mutation (in C/G or A/T positions) was compared to the mean weight of mutable motifs in C/G or A/T positions without mutations in the DNA neighborhood (Supplementary Figure 4) using the *t*-test (2-tailed test) and Monte Carlo test (MC, 1-tailed test) similar to the consensus method, as previously described (Rogozin et al., 2018b). The MC test is based on the random sampling from the group 2. In total, 10,000 groups with size S1 have been generated. The fraction of generated groups with mean weights larger or equal to the mean value of the sample 1 is the P value.

In addition to analyses of the derived mutable motifs in cancer genomes, we performed a control experiment: we randomly shuffled a dataset of sequences surrounding the mutations in the studied target sequences (Supplementary Figures 2, 3) keeping position 6 (the position of mutations) intact. Each sequence was shuffled separately; thus, the overall base composition and the base compositions of each sequence were the same. Weight matrices were derived from these shuffled sequences, and the sampling procedure was repeated 1,000 times.

### Detection of Driver and Non-driver Genes

In this study, we used two independent methods to predict the driver status of cancer mutations: the MutaGene (Goncearenco et al., 2017; Brown et al., 2019) and Chasmplus (Tokheim and Karchin, 2019). These methods showed top performance on a recent benchmarking set (Brown et al., 2019). MutaGene is a probabilistic approach which adjusts the number of mutation recurrences in patients by means of a cancer-type specific background mutation model. The MutaGene driver mutation prediction method has not been explicitly trained on any particular set of mutations. The background models estimate the probability of obtaining a codon substitution from the underlying processes of mutagenesis. We used two MutaGene background models: one was derived from pan-cancer mutational data (“Pancancer” model in MutaGene) whereas the other was constructed directly from the MALY-DE mutational data since this cancer-specific model was not present in the MutaGene database of background models. As a result, two ranking lists of driver mutations were produced for three types of mutation: missense, nonsense and silent. Chasmplus is a machine learning method that was trained using somatic mutations from TCGA. Since no cancer-specific model was available for MALY-DE, we used pan-cancer predictions while running Chasmplus. Then we merged the predictions produced by the three different models/methods and reported only those mutations as drivers which were predicted to be “drivers” or “potential drivers” by MutaGene and had a Chasmplus score cutoff larger than 0.5. Supplementary File 1 shows recurrent driver and passenger mutations.

Predicted driver mutations satisfy at least two of the above-mentioned criteria of driver mutations (Supplementary File 2). Predicted passenger mutations must satisfy all criteria of passenger mutations. Since Chasmplus does not generate predictions for nonsense and silent mutations, only predictions for missense mutations were reported. In addition, some mutations/genes were not reported by Chasmplus because it excluded them from the list of potential cancer driver genes. In this study, we defined driver genes in the following way: a driver gene must have at least one recurrent driver mutation but may also possess recurrent passenger mutations (Supplementary Table 1). Some genes contain only recurrent passenger mutations with frequencies comparable to driver genes. In this study, we defined a non-driver gene operationally as a gene that only contains recurrent mutations that are not associated with the process of tumorigenesis and hence are classified as passenger mutations (Supplementary Table 2).

## Results

### Weight Matrices Are Powerful Descriptors of Mutable Motifs

Weight matrices constitutes a novel technique when applied to the description of preferential mutable motifs. It was shown to be a robust and precise technique to describe AID/APOBEC mutable motifs in cancer cells (Rogozin et al., 2019). The weight matrices include information on the frequency of A, T, G, and C bases in each of the ten positions surrounding the sites of mutation (5 bases downstream and 5 bases upstream). AID, DNA pol η and pol θ are involved in SHM in immunoglobulin genes (Revy et al., 2000; Matsuda et al., 2001; Pavlov et al., 2002; Zan et al., 2005; Neuberger and Rada, 2007; Arana et al., 2008; Bhattacharya et al., 2008), although this role for both polymerases has been questioned (Dörner and Lipsky, 2001; Martomo et al., 2008).

In this study, we started from the construction of weight matrices for both DNA pols. It should be noted that we previously derived weight matrices using collections of mutations induced by AID/APOBEC deaminases in yeast genomes (Rogozin et al., 2019). For human DNA pols η and θ, such collections are not available. Therefore, we used a collection of mutations generated by human pols η and θ during classic gap-filling DNA synthesis *in vitro* (Matsuda et al., 2001; Rogozin et al., 2001; Pavlov et al., 2002) (Supplementary Figures 2, 3). Constructed matrices of nucleotide frequencies are shown in Figure 1A-D (corresponding raw numbers are shown in the Supplementary Figure 1). Pols η and θ exhibit known DNA context features for mutations in A:T sites. W (A or T) or A in the position -1 (Figures 1A,B) was the most prominent feature of A:T mutations produced by pol η and pol θ, accordingly. We cannot exclude the possibility that some other previously undetected positions may contribute to the mutable motifs, for example, a higher frequency of Y (T or C) in position -2 or a lower frequency of G may be additional features of the pol η mutable motif (Figure 1A).

By contrast, pols η and θ exhibit dissimilar DNA context features for mutations at G:C sites. A characteristic feature of pol θ is an elevated frequency of C at position –1 and a lower frequency of C at position –2 (Figure 1C). Thus, pol θ tends to produce more errors in the DCG nucleotide context (D = A or T or G). Pol η appears to have a different DNA mutational context with an excess of C in position +1 (Figure 1D). In general, it is hard to confidently delineate mutable motifs of either DNA polymerase using the consensus approach owing to the lack of objective inclusion criteria for position-specific context features to mutable motifs (Figure 1). Thus, the weight matrix approach, which utilizes information contained in all studied positions, is likely to be a more straightforward way to describe the polymerase η and θ mutable motifs than the consensus approach.

We also compared the nucleotide composition of sequences surrounding positions of mutations (Supplementary Figure 1) for pols η and θ using the χ^2^ test. We found that these pols were significantly different with respect to the DNA sequence context of mutation sites expressed in the form of nucleotide frequency matrices (A:T sites: χ^2^ = 155.0, df = 40, *P* = 1.9 × 10^–15^; G:C sites: χ^2^ = 82.2, df = 40, *P* = 0.00007). Thus, DNA pol η and pol θ differ significantly in terms of the features of the DNA sequence context of mutations. This result is consistent with the different context properties of pols η and θ (Figure 1).

### Footprints of pol η and pol θ Correlate With the Somatic Mutational Spectrum in Many Cancer Types

Previously, we demonstrated using the consensus approach that mutagenesis by AID is likely modulated by the (de)methylation and/or translesion synthesis (TLS) of CpG dinucleotides in follicular lymphomas and many other cancers (Rogozin et al., 2016). Based on analyses of mutations in CpG dinucleotides in skin cancer cells and normal cells, it was also suggested that pol η mutagenesis might also correlate with the methylation of CpG dinucleotides in cancer cells (Rogozin et al., 2018b). The weight matrix approach and the MALY-DE datasets (CpG methylation spectra and somatic mutations, see Materials and Methods) allow us to perform further analyses of the role of AID and error-prone polymerases in mutagenesis, and to see how it is affected by (de)methylation.

**FIGURE 3:**
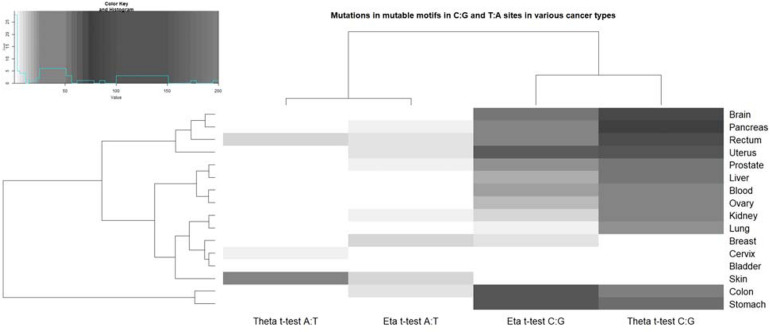
Correlation of pol η (eta) and θ (theta) mutable motifs and the sequence context of somatic mutations. For the actual data, see Supplementary Tables 3, 4. The intensities of the gray color correspond to the *t*-test values (Supplementary Tables 3, 4). The unweighted pair group method, with arithmetic mean (UPGMA) clustering of ratio values for the pol η and θ footprints and tissues, is shown to the left and top. The upper left panel shows the distribution of the studied *t*-test values and correspondence of the *t*-test values and color intensity (the darker colors correspond to the higher correlation values). A similar plot of ratio values (the ratio being the mean weight of mutated sites divided by the mean weight of non-mutated sites) is shown in the Supplementary Figure 5.

We examined the correlation between the nucleotide sequence context of somatic mutations in cancers and pol η and pol θ mutable motifs found after *in vitro* DNA synthesis. A correlation was inferred when the results of two statistical tests (Monte Carlo test and *t*-test) were significant at *P* < 0.05. AID has already been studied using the consensus motif WRC/GYW and weight matrices and has been shown to be one of the most ubiquitous contributors to mutations in various cancer types according to its characteristic mutable motif (the AID weight matrix) (Rogozin et al., 2019). Analysis of pol-generated mutations in G:C sites revealed that both mutation motifs are almost universally correlated with the nucleotide context of somatic mutations in G:C sites (Figure 3). Similar analysis of A:T site mutations also revealed correlations for pol η. A significant correlation with pol θ was documented only for a few cancer cases. This difference may reflect a more specialized role for pol θ in DNA transactions on methylated CpG’s (Wood and Doublié, 2016; Brambati et al., 2020). It is also possible that pol θ is expressed in only a few cancers. Pol η probably plays a more widespread, although not particularly pronounced, role in causing mutations in cancer according to its characteristic weight matrix in various cancer types; this is consistent with our previous study where we used the consensus sequence WA (Rogozin et al., 2018b).

### Control Experiments

The *in vitro* collections of mutations that were used to reconstruct weight matrices for pol η and pol θ (Matsuda et al., 2001; Arana et al., 2008) are relatively small (Supplementary Figures 2, 3). Thus, control experiments were important to analyze the quality of the derived weight matrices. We previously demonstrated that analyses of the association between the matrices of shuffled sites of mutation and the nucleotide context of somatic mutation in various cancer cell types is a reliable approach to estimate the impact of the accuracy of association prediction (Rogozin et al., 2019). Analysis of 16 types of cancer (Supplementary Table 5) suggested that the AID weight matrix is less prone to errors of prediction compared to pol η/pol θ (Supplementary Table 5). Only a few types of cancer have a low level of prediction errors. Fortunately, for our study of MALY-DE sets, “Blood” tissue, GCB lymphomas (from the COSMIC database) and MALY_DE malignant lymphomas have extremely low rates of false prediction (Supplementary Table 5). Therefore, we opted to use the derived matrices for further analysis of the MALY-DE datasets.

Analysis of somatic mutations in immunoglobulin genes can be used to estimate the prediction accuracy because the context of mutations in human immunoglobulin genes is known to correlate strongly with AID and pol η mutable motifs (Matsuda et al., 2001). Thus, these mutations can be used as a control dataset as performed previously (Rogozin et al., 2019). A significant association between the AID mutable motif and mutations was found in all three studied somatic mutation datasets (Milstein et al., 1998; Mayorov et al., 2005; Table 1), confirming that the AID weight matrix is a reliable descriptor of AID-induced mutagenesis. The pol η weight matrices revealed a significant association for all studied cases except xeroderma pigmentosum variant (XPV) patients where pol η is inactive (Table 1; Mayorov et al., 2005). Pol θ matrices yielded significant results for some studied cases (Table 1). This is consistent with the hypothesis that pol θ is also involved in SHM (Arana et al., 2008). The results of both control experiments suggested that the weight matrix technique approach is adequate to study the mutational spectra of DNA polymerases.

**TABLE 1:** Correlation between the sequence context of somatic mutations and mutable motifs in fragments of human immunoglobulin genes.

Locus	Test	Number of Mutations	AID/G:C	Pol η/G:C	Pol θ/G:C	Number of Mutations	Pol η/A:T	Pol θ/A:T
V_*H*_26	Ratio	583	1.208	1.027	1.091	351	1.082	0.979
	*t*-test		13.1*	NSE	5.9*		5.3*	NSE
	MC test		<0.001	0.004	<0.001		<0.001	0.699
J_*H*_4 intron, control individuals	Ratio	177	1.341	1.05	1.029	95	1.041	1.032
	*t*-test		12.3*	2.8*	NSE		2.4*	2.2*
	MC test		<0.001	0.002	0.106		0.004	0.011
J_*H*_4 intron, XP-V patients	Ratio	227	1.278	1.009	1.011	25	0.957	0.98
	*t*-test		9.9*	NSE	NSE		NSE	NSE
	MC test		<0.001	0.329	0.061		0.776	0.67

*“Ratio” is the mean weight of mutated sites divided by the mean weight of non-mutated sites.*

NSE (no significant excess) indicates the absence of a significant excess of mutations in mutable motifs, suggesting there to be no association between mutagenesis and mutable motifs. The significance of any excess was measured using the Student *t* and Monte Carlo (MC) tests. The asterisk (*) denotes that the corresponding *P* < 0.01; this is a conservative estimate of the critical overall value of the *t*-test having allowed for multiple testing by means of the Bonferroni correction (5 comparisons).

### Analysis of Driver and Non-driver Genes

Analysis of driver/passenger mutations is known to be powerful approach in cancer genomics and can even be diagnostic of various cancers (Goncearenco et al., 2017; Brown et al., 2019; Tokheim and Karchin, 2019; Dietlein et al., 2020). We derived lists of recurrent driver and non-driver mutations using three computational approaches (see section “Materials and Methods”). We define driver genes as those genes, which accumulate recurrent driver mutations, but which may also possess recurrent passenger mutations (Supplementary Tables 1). Some genes contain only recurrent passenger mutations with frequencies comparable to driver genes; in this study, we defined a non-driver gene operationally as a gene that only contains recurrent passenger mutations (Supplementary Table 2).

Final lists of operationally defined driver and non-driver genes are shown in Supplementary Tables 1, 2 (we used the ENSEMBL IDs, as recommended by the DAVID Bioinformatics Resources web site, https://david.ncifcrf.gov/). The total numbers of driver and non-driver genes are 134 and 210, respectively. We performed pathway/keyword enrichment analyses (Luque-Baena et al., 2014; Wang et al., 2014; Soldatos et al., 2015) using the DAVID web site (Jiao et al., 2012). Results are shown in the Supplementary Table 6. Keywords “methylation,” “nuclear chromatin,” and numerous pathways/terms associated with various types of cancer are consistent with properties of GCB lymphomas (Green et al., 2015; Rogozin et al., 2016). The KEGG pathway “pathways in cancer” (*P* = 0.025) is another important descriptor of the driver gene list (Supplementary Table 6). In general, the driver gene set appears to be highly informative and contains many features expected for cancer-related genes (Green et al., 2015) (Supplementary Table 6). By contrast, analysis of non-driver genes yielded only a few significant results with no obvious functional associations with cancer (Supplementary Table 6).

**FIGURE 4:**
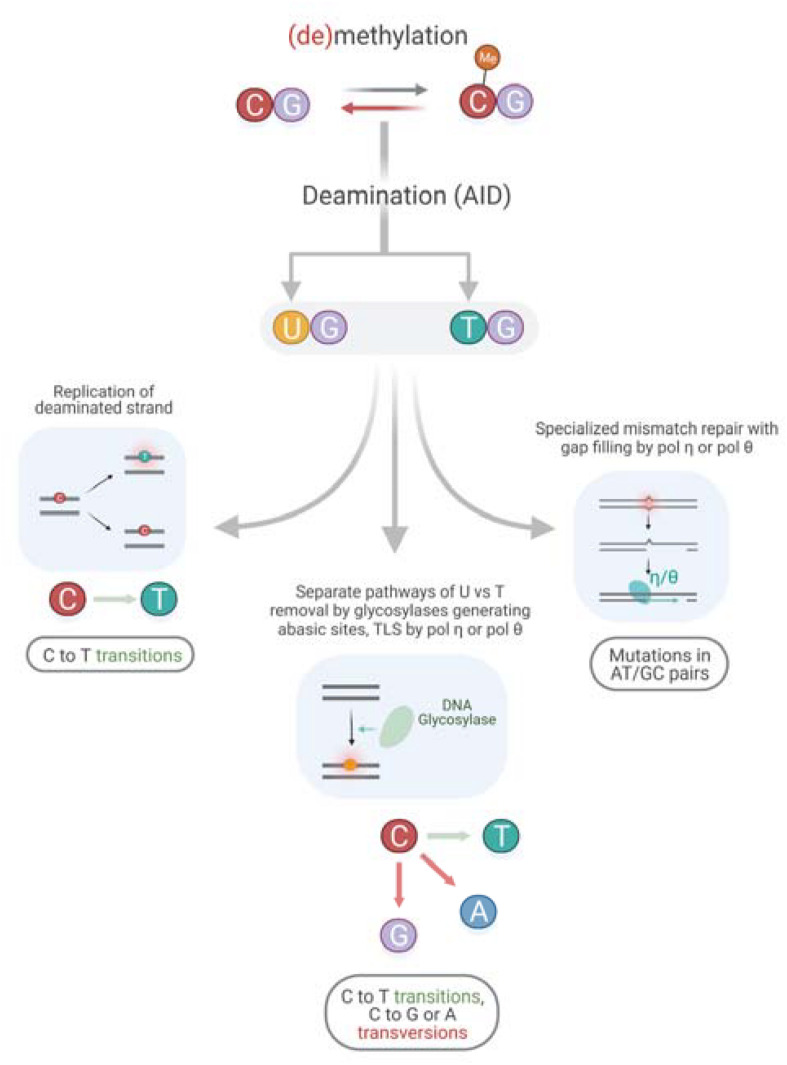
Putative mechanism of an interplay between AID and TLS polymerases.

There is a significant association of the AID mutable motif with somatic mutations in all genes, as well as in driver and non-driver genes (Table 2) suggesting that AID plays an important role in mutagenesis in cancer genomes; there are several pathways that can explain this process (Figure 4). Analysis of association between pols η and θ mutable motifs and somatic mutations detected a difference between driver and non-driver genes: mutable motifs in G:C pairs of pols η and θ correlate with somatic mutations in non-driver genes only. There was no correlation with pol η mutations at A:T pairs, whereas the pattern of somatic mutation correlated with pol θ at A:T sites both in driver and non-driver genes (Table 2). These observations indicate that the contribution of different pathways of generation of mutations in cancers (Figure 4) is distinct for AID, pols η and pol θ.

**TABLE 2:** Correlation between mutable motifs and the sequence context of somatic mutations in driver and non-driver genes.

Group of genes	Test	Number of G:C mutations	AID/G:C	Pol η/G:C	Pol θ/G:C	Number of A:T mutations	Pol η/A:T	Pol θ/A:T
All genes	Ratio	137,775	1.021	1.005	1.091	145,768	0.992	1.011
	*t*-test		23.4*	7.2*	23.0*		NSE	15.8*
	MC test		<0.001	<0.001	<0.001		1	<0.001
Drivers	Ratio	4,246	1.107	1.001	1.007	3,918	0.98	1.032
	*t*-test		20.0*	NSE	NSE		NSE	7.8*
	MC test		<0.001	0.346	0.037		1	<0.001
Non-drivers	Ratio	3,553	1.079	1.059	1.057	2,793	0.995	1.045
	*t*-test		14.2*	13.8*	11.7*		NSE	8.9*
	MC test		<0.001	<0.001	<0.001		0.874	<0.001

*“Ratio” is the mean weight of mutated sites divided by the mean weight of non-mutated sites.*

NSE (no significant excess) indicates the absence of a significant excess of mutations in mutable motifs suggesting there to be no association between mutagenesis and mutable motifs. The significance of any excess was measured using the Student *t* and Monte Carlo (MC) tests. The asterisk (*) denotes that the corresponding *P* < 0.01; this is a conservative estimate of the critical overall value of the *t*-test having allowed for multiple testing by means of the Bonferroni correction (5 comparisons).

Another important feature of driver genes is a higher frequency of mutations at G:C nucleotides (4,246 and 3,918 in G:C and A:T, accordingly) compared to all other genes (137,775 – 4,246 = 133,529 and 145,786 – 3,918 = 141,868 in G:C and A:T, accordingly, Table 2) (*P* < 0.0001 according to the two-tailed Fisher’s exact test).^3^ A similar trend was observed for non-driver genes (Table 2, *P* < 0.0001). This may be explained by a leading role for AID/APOBEC enzyme(s) that preferentially participate in mutagenesis pathways in G:C nucleotides; AID is one such enzyme (Figure 4).

### Patient-Specific Analysis of Somatic Mutations and Methylation

We analyzed the significance of association between AID/pol mutable motifs and the sequence context of somatic mutations for each sample (Supplementary Table 7). The results suggested that all studied samples have a significant association between AID/pols mutable motifs and mutation (Supplementary Table 7). The *t*-test values were similar to those in the merged dataset (Supplementary Table 7 and Table 2). For example, *t*-test values for AID vary from 4.2 to 35.8 (Supplementary Table 7), this value for the merged dataset was estimated as 23.4 (Table 2).

We also analyzed the level of methylation in CpG sites for driver and non-driver genes for each sample separately. We derived profiles of methylation (methylation levels, positions, and chromosomes) across driver and non-driver genes separately. After that, pairwise correlation coefficients (Pearson’s linear correlation coefficients CC) were estimated across all studied samples. All correlation coefficients were larger than 0.9 (the significance level < 0.001). Plots of pairwise CC values are shown in the Supplementary Figures 6, 7; these plots appear homogeneous (no blocks of “high” and “low” CC values that are adjacent in data matrices) (Supplementary Figures 6, 7).

These results suggest that studied patient-specific associations of mutable motifs with somatic mutations as well as patterns of methylation are homogeneous for driver and non-driver genes. Thus, we pooled patient-specific samples into merged datasets of somatic mutations and methylation profiles. This procedure is especially important for the analysis of small datasets that will be described below.

### Analysis of DNA Methylation Patterns of Driver and Non-driver Genes Using Weight Matrices

The average methylation level of driver and non-driver genes was found to be approximately the same: ∼78% for both sets of genes (all CpG dinucleotides in driver and non-driver genes were computationally analyzed using the MALY-DE dataset). Analysis of methylation in mutable motifs was performed using the threshold methylation values 25 and 75%. These two values were chosen arbitrarily, values of 75 (close to the average methylation level) and higher correspond to heavily methylated CpG sites. The value 25% and smaller correspond to CpG sites that are close to the unmethylated state. Thus, values 25 and 75% reflect a dramatically different methylation status for CpG sites in the studied sets of genes (Figure 2).

Let us illustrate the logic of combined analysis of methylation in mutable motifs using an example from Table 3A. For the set of driver genes and the threshold methylation value = 25%, average weights of AID mutable motifs for subsets of CpG sites with methylation values smaller than and greater than the threshold = 25% were 57.8 and 56.4, respectively. The ratio of these values is 1.025 (57.8/56.4 = 1.025) and is shown in Table 3A. This difference is statistically significant, albeit not dramatically so (Table 3). Average weights of AID mutable motifs for non-driver genes below and above the threshold = 25% are 57.7 and 56.2, accordingly. The ratio is 1.027, and this difference is also statistically significant (Table 3). These results suggest that a high frequency of AID-mutable motifs is associated with lower methylation levels in driver and non-driver genes. For pol η and θ, no significant differences were detected for both driver and non-driver genes (Table 3A), suggesting that the global level of methylation of CpG sites of driver and non-driver genes for the threshold methylation level = 25% may not interfere with mutagenesis by pols η and θ.

**TABLE 3:** Analysis of methylation in CpG sites that overlap with pols η and θ mutable motifs.

Group of genes	Number of CpG sites below and above the threshold	Tests	AID	Pol η	Pol θ
**A. Levels of methylation in CpG sites that overlap with mutable motifs, with the threshold value = 25%**					
Driver		Ratio	1.025	0.997	0.994
	2,867	*t*-test	3.2*	NSE	NSE
	149,480	MC test	<0.001	0.772	0.95
Non-driver		Ratio	1.027	0.993	0.985
	5,558	*t*-test	5.4*	NSE	NSE
	239,220	MC test	<0.001	0.989	0.989
**B. Levels of methylation in CpG sites that overlap with mutable motifs, with the threshold value = 75%**					
Driver		Ratio	1.004	1.009	1.021
	96,917	*t*-test	NSE	7.9*	20.4*
	51,290	MC test	0.433	<0.001	<0.001
Non-driver		Ratio	1.007	1.009	1.023
	155,205	*t*-test	4.5*	9.8*	28.6*
	89,573	MC test	<0.001	<0.001	<0.001

“Ratio” is the mean weight of mutable motifs in CpG sites with methylation values below (or above) the threshold divided by the mean weight of mutable motifs in CpG sites with methylation values above (or below) the threshold (25 or 75%, respectively) (a schematic representation of this analysis is shown in Figure 2. NSE (no significant excess) indicates the absence of any significant excess suggesting there to be no association between methylation and mutable motifs. The significance of an excess was measured using the Student *t* and Monte Carlo (MC) tests. The asterisk (*) denotes that the corresponding *P* < 0.01; this is a conservative estimate of the critical overall value of the *t*-test having allowed for multiple testing by means of the Bonferroni correction (3 comparisons).

For the threshold methylation value = 75%, we observed to some extent the opposite trend. For example, the average weights of AID-mutable motifs for driver genes greater and smaller than 75% were 56.9 and 56.7, respectively. The ratio of these values is 1.004 (56.9/56.7 = 1.004) (Table 3B). This difference is not statistically significant (Table 3B). The ratio is also relatively low for the non-driver gene set although it is significant (Table 3B). Mutable motifs for both studied DNA polymerases appear to be associated with the methylation level for this threshold (heavily methylated CpG sites). These results suggest that the global level of methylation in driver genes for the heavily methylated positions may be affected by pol η and pol θ transactions on methylated CpG’s but not AID transactions. The methylation levels of non-driver genes may be affected by all studied enzymes (Table 3B).

### Analysis of Somatic Mutations in CpG Sites in Driver and Non-driver Genes

We analyzed the level of methylation in CpG sites that coincide with positions of somatic mutation. This dataset is much smaller compared to all methylated CpG’s (the previous section). It should be noted that the studied sets are small. However, they are still amenable to statistical analysis using the threshold = 75% (Table 4, heavily methylated CpG sites). Unfortunately, the number of mutations for the threshold = 25% (CpG sites that are close to the unmethylated state) was too small for statistical analysis: the number of mutated sites with methylation levels below 25% is 0 and 3 for driver and non-driver genes, accordingly. Thus, we did not use the threshold value 25% but instead used the threshold value 75% only.

**TABLE 4:** Levels of methylation in positions of somatic mutation in CpG sites, the threshold value = 75%.

Group of genes	Number of mutations in CpGs sites above and below the threshold	Tests	AID	Pol η	Pol θ
Driver		Ratio	1.111	1.136	1.046
	249	*t*-test	2.9*	7.8*	NSE
	52	MC test	0.004	<0.001	0.009
Non-driver		Ratio	1.015	1.125	1.061
	390	*t*-test	NSE	7.3*	3.7*
	264	MC test	0.222	<0.001	<0.001

“Ratio” is the mean weight of mutated CpG sites above the methylation threshold divided by the mean weight of mutated sites below the threshold (a schematic representation of this analysis is shown in the Supplementary Figure 8). NSE (no significant excess) indicates the absence of any significant differences between these sets suggesting there to be no association between mutagenesis and motifs in the CpG sites. The significance of any excess was measured using the Student *t* and Monte Carlo (MC) tests. The asterisk (*) denotes that the corresponding *P* < 0.01; this is a conservative estimate of the critical overall value of the *t*-test having allowed for multiple testing by means of the Bonferroni correction (3 comparisons).

The first result obtained is that the fraction of mutated CpG sites with methylation values below the threshold 75% is dramatically different for driver genes (52/(52+249) = 0.17, Table 4, the second column) and non-driver genes (0.40, Table 4, the second column). This difference is statistically significant (P < 0.0001 according to the two-tailed Fisher’s exact test). Thus, CpG sites with somatic mutations in driver genes tend to have higher methylation values compared to non-driver genes.

The second interesting result is the significant correlation of AID, pol η and pol θ with mutation positions having a lower methylation level (below 75%) (Table 4). The correlation of the AID motif presence and mutation is more pronounced for driver genes, indicating that AID-induced mutagenesis is likely to be associated with heavily methylated CpG dinucleotides. Pol η has a role in CpG mutagenesis for both sets of genes whereas pol θ is likely to be largely involved in the mutagenesis of non-driver genes (Table 4). Thus, it is likely that methylation levels influence mutagenesis pathways in CpG sites through the action of all the studied enzymes, although the individual impact of studied enzymes may be different for driver and non-driver genes (for example, AID, Table 4). It is likely to depend on various factors including gene expression. This will be discussed in the next section.

### Analysis of Expression of Driver and Non-driver Genes

**FIGURE 5:**
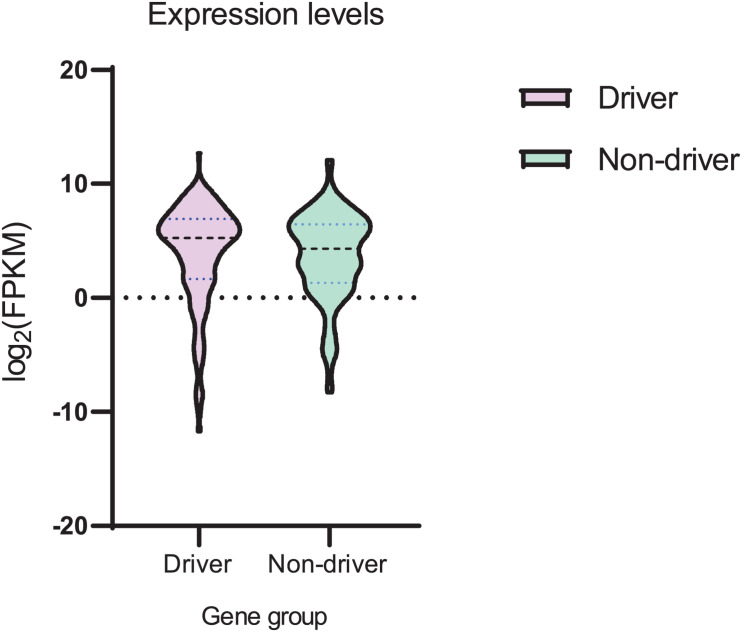
Violin plot of mRNA expression (FPKM values) for sets of driver and non-driver genes. Log_2_ transformation was used.

We analyzed the expression levels (FPMK values) for both sets of genes (Supplementary Tables 1, 2). Analysis of mean and variance (Figure 5 and Supplementary Table 8) suggested that mean values were not substantially different. However, the variance of expression values observed in the set of driver genes was larger as compared to the set of non-driver genes (Supplementary Table 8). The difference between mean values (Supplementary Table 8) was not statistically significant (*t*-test *P* value = 0.086), whereas the difference between variance values (Supplementary Table 8) was statistically significant (*F*-test *P* value = 0.007).

## Discussion

Some results of this study seem to be counterintuitive. For example, the AID mutable motif would appear to correlate with the context of somatic mutations in heavily methylated CpG’s for driver genes only (Table 4). It is hard to determine the factors that are responsible for this difference. For example, variability of gene expression is significantly higher for driver genes (Figure 5). This may be associated with the differential regulation of expression of driver genes in different patients or methylation levels. Copy number variation of driver genes (Loohuis et al., 2014; Cheng et al., 2016) may cause problems for precise estimates of CpG methylation levels.

AID and DNA polymerases η/θ are known to participate in somatic hypermutation of immunoglobulin genes (Matsuda et al., 2001; Casali et al., 2006; Neuberger and Rada, 2007; Bhattacharya et al., 2008). In addition, it has been suggested that AID and pol η are likely to contribute to a lowering methylation levels of CpG dinucleotides in cancer cells (Rogozin et al., 2018b). Thus, we focused this study on AID and pols η/θ employing the weight matrix technique and mutation/methylation profiles. Our results suggest that AID and pols η/θ combine to generate footprint mutations in B-cell derived lymphomas and other cancers. It was reported that methylation substantially reduces the rate of APOBEC-induced mutations in CpG dinucleotides (Seplyarskiy et al., 2016). For this reason, we did not include other members of the AID/APOBEC superfamily in the current study.

The advantage of the weight matrix approach is that it is a unified computational technique that allows an accurate and objective comparison of the mutational contribution of various mutator enzymes under the same experimental conditions and for the same datasets. We confirm that while the mutational footprints of DNA polymerases η and θ are prominent in some cancers, mutable motifs characteristic of the humoral immune response somatic hypermutation machine, AID, is likely to be the most widespread feature of somatic mutational spectra attributed to any enzyme in cancer genomes (Rogozin et al., 2018b, 2019). It is important to note that the suggested technique does not depend on expert opinion as to the exact consensus sequences, and therefore objectively represents mutable motifs.

We derived matrices for A:T and G:C residues. However, the ratio of A:T to G:C mutations is variable (Supplementary Figure 1). For example, it known that Pol η mutates G residues at a lower frequency than A residues. However, two matrices (G:C and A:T residues, Figure 1) for the two motifs were used independently (Figure 3). We would like to develop a probabilistic model that integrates two matrices in one model. However, this approach has never been attempted before in this context and would require further investigation.

It is not possible to delineate the exact mechanism of the interplay between AID and DNA polymerases. It may be replication of the deaminated strand, separate pathways of U vs. T removal by glycosylases generating abasic sites followed by TLS by pol η or pol θ, and/or specialized mismatch repair with gap filling by pol η or pol θ (Figure 4) (Pilzecker and Jacobs, 2019). Unfortunately, precise mechanisms have not been clearly defined even for mutagenesis of immunoglobulin genes, with attempts to define those mechanisms having been ongoing for over 20 years.

A high rate of prediction errors for many types of cancer (Supplementary Table 5) is likely to be due to the small mutational spectra available for DNA polymerase η and θ (Supplementary Figures 2, 3). Larger sets of mutations are likely to improve the quality of prediction. We can nevertheless infer that some types of cancer, including GCB lymphomas, do not have a noticeable rate of false positives (Supplementary Table 5). We applied all weight matrices to study mutable motifs and methylation in the MALY-DE datasets and demonstrated that mutable motifs correlate with CpG dinucleotides and their methylation status. Another methodological problem is the small number of MALY-DE samples (26 samples) which may cause problems for the prediction of driver and passenger mutations. These problems are one of several possible explanations as to why differences between driver and non-driver genes are subtle (albeit significant) (Tables 2-4). However, it is likely that these differences are responsible for the major difference observed between the expression of driver and non-driver genes (Figure 5). The much large variance observed for driver genes may be the result of greater (de)methylation of driver gene sequences causing substantial variability of mRNA expression across patients (Figure 5).

Sophisticated classification approaches (prediction of mutational signatures) have been developed to extract the most prominent signatures from a complex mix of mutational spectra resulting from the action of a variety of mutagens, both exogenous and endogenous, operating during tumor evolution (Petljak and Alexandrov, 2016; Rahbari et al., 2016; Goncearenco et al., 2017; Rogozin et al., 2018c; Alexandrov et al., 2020). Both driver and passenger mutations have been used in the analysis without any attempt to analyze them separately. In this study, we analyzed driver and non-driver genes separately and detected significant differences in the relationship between mutable motifs and mutations with the methylation/demethylation status of driver and non-driver genes (Tables 3 and 4). It is not that easy to interpret these differences because the role of methylated CpG dinucleotides in exons is not yet fully understood (Neri et al., 2017). It has been suggested that changes in intragenic DNA methylation is important in several human diseases including syndromic and sporadic forms of various neurological disorders that involve methylation defects, including Rett syndrome, Prader–Willi and Angelman syndromes, and others, suggesting that the differential (de)methylation of genes may underpin one aspect of various neurological disorders (Dunaway et al., 2016; Rogozin et al., 2018a; Scandaglia and Barco, 2019). Such differential methylation may be caused by differences in (de)methylation processes in somatic/germ cells (Shanak and Helms, 2020). Moreover, several studies of likely deleterious mutations have observed that genes controlling methylation status, chromatin accessibility or remodeling (and hence gene expression) are enriched for genes with recurrent mutations (Geschwind and State, 2015; Sanders et al., 2015; Geisheker et al., 2017).

The difference in AID and polymerase properties (Tables 3, 4) for driver and non-driver genes is consistent with the participation of different mechanisms of mutagenesis and (de)methylation processes (Figure 4) on non-methylated and methylated DNA. The observed differences between driver and non-driver genes associated with somatic mutations in driver genes (Tables 3, 4) are likely to cause changes in gene expression (Figure 5) that then trigger cancer initiation and/or progression. This is not surprising if we consider that chromatin modification pathways (Supplementary Table 6) as well as the observed changes in CpG methylation levels (Tables 3, 4) are likely to cause changes in the expression levels of driver genes that could affect both cancer initiation and/or progression.

## Data Availability Statement

Publicly available datasets were analyzed in this study. This data can be found here: https://dcc.icgc.org/projects/MALY-DE; https://cancer.sanger.ac.uk.

## Author Contributions

IBR, AR-L, KT, KC-C, AL, LP, and ES: formal analysis. All authors: investigation.

## Conflict of Interest

The authors declare that the research was conducted in the absence of any commercial or financial relationships that could be construed as a potential conflict of interest.

## Footnotes

1 https://cancer.sanger.ac.uk

2 https://dcc.icgc.org/projects/MALY-DE

3 www.graphpad.com/quickcalcs/contingency1.cfm
