# Supplementary material for: DNA Methylation, Deamination, and Translesion Synthesis Combine to Generate Footprint Mutations in Cancer Driver Genes in B-Cell Derived Lymphomas and Other Cancers
Source: Front Genet. 2021 May 19;12:671866. doi: 10.3389/fgene.2021.671866 (PMC8170131; doi:10.3389/fgene.2021.671866)

**Supplementary Materials**

**DNA methylation, deamination and translesion synthesis combine to generate footprint mutations in cancer driver genes in B-cell derived lymphomas and other cancers**

**Supplementary Table S1. List of driver genes and corresponding mean and variance of expression levels (FPKM) across 26 patients.**

Gene name mean variance

ENSG00000117318 88.8097 8391.1640

ENSG00000112902 14.9833 264.6556

ENSG00000084636 29.8132 3748.5940

ENSG00000104723 8.4185 32.1109

ENSG00000115652 39.5469 344.5692

ENSG00000168944 78.8358 975.3209

ENSG00000171209 .0602 .0059

ENSG00000109084 31.5416 253.7073

ENSG00000144452 4.4860 8.3889

ENSG00000075275 64.6227 1085.7240

ENSG00000161405 83.6811 1550.6470

ENSG00000179869 3.2274 63.1910

ENSG00000075624 6772.6360 7818099.0000

ENSG00000150394 .8577 .1806

ENSG00000215193 50.3035 78.7689

ENSG00000136997 286.6876 106725.5000

ENSG00000125409 .2686 .0519

ENSG00000170234 56.6067 55.8462

ENSG00000138193 11.4735 6.8901

ENSG00000175097 3.2206 115.7028

ENSG00000125686 109.9446 755.0569

ENSG00000163406 56.1048 1258.4770

ENSG00000145362 177.0673 7278.6000

ENSG00000198104 .0026 .0000

ENSG00000178607 20.7277 302.4223

ENSG00000120549 17.6199 99.8933

ENSG00000116954 38.2899 109.6477

ENSG00000168530 .0506 .0017

ENSG00000169488 .0034 .0001

ENSG00000127914 141.5064 5494.3640

ENSG00000064763 30.0544 175.7585

ENSG00000171914 7.9272 30.5254

ENSG00000184304 3.0612 4.3863

ENSG00000085185 10.2510 29.4533

ENSG00000141510 159.9300 4490.9260

ENSG00000148386 .0254 .0019

ENSG00000138650 .1246 .0124

ENSG00000127481 351.9393 14291.9000

ENSG00000135144 327.8380 36475.8200

ENSG00000145675 85.9121 1265.1570

ENSG00000255223 .0003 .0000

ENSG00000149923 157.9395 1794.4910

ENSG00000198171 37.9427 55.4320

ENSG00000110536 49.4751 248.1391

ENSG00000162522 2.7586 9.5244

ENSG00000115998 219.0992 6071.8170

ENSG00000107736 53.0623 1349.7580

ENSG00000157873 150.2094 14460.8200

ENSG00000178951 39.4858 107.3366

ENSG00000204946 56.3628 566.5106

ENSG00000135334 80.4638 801.8282

ENSG00000105877 50.5685 484.7126

ENSG00000153820 .4666 .1617

ENSG00000149972 9.7230 7.8528

ENSG00000175161 2.7146 2.3697

ENSG00000172071 118.4903 2000.8730

ENSG00000028277 341.5431 38098.4600

ENSG00000182447 .0137 .0002

ENSG00000127616 535.0883 122082.5000

ENSG00000182197 27.8858 208.9541

ENSG00000147324 91.4854 3695.7500

ENSG00000182836 2.2483 9.1759

ENSG00000169813 347.2354 10336.0100

ENSG00000171791 122.9759 23064.2500

ENSG00000135679 226.0374 19391.9000

ENSG00000083857 13.9704 247.4364

ENSG00000149311 355.3136 47526.4200

ENSG00000071564 772.9116 241346.1000

ENSG00000143344 40.4101 1661.0490

ENSG00000137936 37.3351 1607.2870

ENSG00000218336 5.1580 20.2460

ENSG00000137497 457.3446 12422.1600

ENSG00000186212 .0244 .0019

ENSG00000058335 14.8998 190.1614

ENSG00000111642 540.3575 27160.1700

ENSG00000129347 122.5863 1283.1740

ENSG00000021852 .0845 .0060

ENSG00000104369 1.9499 14.6327

ENSG00000100106 70.6132 423.8156

ENSG00000138709 18.1220 62.4958

ENSG00000184634 99.8658 1390.2960

ENSG00000166342 5.9420 407.5891

ENSG00000138670 57.3591 1243.1280

ENSG00000215421 26.6265 26.0755

ENSG00000168298 .9471 .9591

ENSG00000091157 36.4617 80.1570

ENSG00000163939 158.1751 3810.9550

ENSG00000071553 96.3798 2309.9260

ENSG00000104312 45.2444 401.9473

ENSG00000114770 42.6997 306.6271

ENSG00000168421 284.3458 22684.2700

ENSG00000196092 533.9325 96457.6600

ENSG00000067560 725.5257 22557.4300

ENSG00000198734 9.0969 203.0947

ENSG00000121879 79.1009 390.1123

ENSG00000130508 29.9114 819.6361

ENSG00000147416 129.1586 3627.4170

ENSG00000109756 34.7807 272.2620

ENSG00000143105 .0033 .0000

ENSG00000182621 11.0877 179.3913

ENSG00000188176 .6377 2.3654

ENSG00000179344 586.4301 182417.5000

ENSG00000233822 1.4947 1.0605

ENSG00000124635 2.8258 11.4042

ENSG00000124942 258.1852 104558.7000

ENSG00000039650 78.6209 2051.5280

ENSG00000186354 .9083 .6309

ENSG00000050628 2.4050 7.6063

ENSG00000074527 3.9612 26.2779

ENSG00000162408 42.2342 182.5803

ENSG00000140564 55.4913 1492.7370

ENSG00000003436 19.4683 422.5746

ENSG00000182162 .0000 .0000

ENSG00000142065 13.7268 62.1910

ENSG00000185985 .5575 .4549

ENSG00000106278 .7156 1.8722

ENSG00000121871 .0447 .0026

ENSG00000166888 589.8515 103126.9000

ENSG00000154654 1.8743 1.5053

ENSG00000164796 57.2506 343.5894

ENSG00000163539 119.7879 1217.1210

ENSG00000065526 200.3710 7957.6550

ENSG00000151834 .6290 .1327

ENSG00000136878 73.7831 1121.7140

ENSG00000124486 199.1426 3592.0150

ENSG00000172489 .0012 .0000

ENSG00000198793 145.8514 1434.4420

ENSG00000052841 130.5034 1538.5900

ENSG00000050438 17.1854 408.8041

ENSG00000111057 3.0687 22.7487

ENSG00000134982 55.0035 198.3359

ENSG00000012061 198.5452 41149.3200

ENSG00000124391 .2647 .1255

ENSG00000163737 .4333 2.3449

**Supplementary Table S2. List of non-driver genes and corresponding mean and variance of expression levels (FPKM) across 26 patients.**

Gene name mean variance

ENSG00000142192 127.0531 10349.8600

ENSG00000162676 14.1019 97.7193

ENSG00000146839 1.4079 7.5056

ENSG00000160949 57.2216 1195.9950

ENSG00000065534 51.1063 5995.9390

ENSG00000148634 124.0384 2306.3860

ENSG00000136160 10.6781 131.3169

ENSG00000126247 288.8461 8914.1010

ENSG00000160688 56.1844 202.4997

ENSG00000167996 763.1808 563911.0000

ENSG00000151240 54.2447 2113.1490

ENSG00000213999 293.1811 42616.8900

ENSG00000234745 3263.2360 4737292.0000

ENSG00000170421 7.0971 168.4019

ENSG00000135870 49.8087 355.9715

ENSG00000182256 2.1064 5.3850

ENSG00000168438 70.1869 406.7942

ENSG00000186716 140.7908 3424.9580

ENSG00000181885 1.5104 1.4674

ENSG00000106785 127.8934 1594.4980

ENSG00000126464 62.3963 390.0819

ENSG00000204920 11.9205 56.9092

ENSG00000160460 2.0086 3.1757

ENSG00000154678 3.8185 11.8384

ENSG00000179981 18.1004 63.8538

ENSG00000158488 3.2638 40.9797

ENSG00000196344 .0372 .0006

ENSG00000109794 2.3821 5.0236

ENSG00000106113 1.2211 .4180

ENSG00000128578 10.5355 23.6109

ENSG00000138316 5.2039 22.2849

ENSG00000101323 .1399 .0078

ENSG00000108559 295.1587 6052.9240

ENSG00000171773 .0503 .0030

ENSG00000173801 389.5156 62063.3900

ENSG00000127526 50.4748 122.8640

ENSG00000233436 .3526 .0416

ENSG00000186642 7.7608 90.8830

ENSG00000108506 44.1528 152.0129

ENSG00000140961 1.2344 .9235

ENSG00000131196 146.2744 3759.3770

ENSG00000035141 52.6702 375.2318

ENSG00000185630 9.8982 132.3424

ENSG00000147724 1.0655 1.0359

ENSG00000042832 98.7840 7251.2640

ENSG00000168631 .1895 .0385

ENSG00000167104 .0399 .0016

ENSG00000128833 19.9357 60.7010

ENSG00000196159 4.1119 28.3836

ENSG00000118564 124.5039 1483.1820

ENSG00000146587 34.8691 75.2363

ENSG00000180481 .6686 .3200

ENSG00000196126 1121.6510 464061.3000

ENSG00000185008 89.1434 1085.1230

ENSG00000124003 .1580 .0121

ENSG00000140682 8.0950 46.1206

ENSG00000146918 135.2603 6733.9390

ENSG00000166669 53.3757 681.7703

ENSG00000100504 22.6421 879.9482

ENSG00000198482 21.7481 75.1884

ENSG00000173908 .0139 .0003

ENSG00000162769 20.0487 27.6951

ENSG00000204560 82.3354 235.3753

ENSG00000172236 6.9457 184.0351

ENSG00000102119 79.4997 153.7123

ENSG00000059915 6.1615 6.9687

ENSG00000134571 2.4827 6.2289

ENSG00000127081 7.7158 7.1309

ENSG00000090273 54.1064 418.6224

ENSG00000138767 91.1232 1139.2850

ENSG00000161180 .4599 .0790

ENSG00000026950 93.8956 5724.4020

ENSG00000101438 .0407 .0024

ENSG00000125813 .0394 .0038

ENSG00000125618 38.4241 552.8815

ENSG00000156103 1.4500 1.5624

ENSG00000231852 .8789 2.0174

ENSG00000188641 61.9938 759.5113

ENSG00000136002 3.8246 15.9362

ENSG00000140067 .0851 .0027

ENSG00000108950 19.0208 408.3891

ENSG00000118515 166.1102 28946.9300

ENSG00000184164 43.5330 139.8835

ENSG00000121440 4.0532 21.7427

ENSG00000113719 168.6084 1475.3830

ENSG00000174669 22.8937 292.2443

ENSG00000123165 .0046 .0001

ENSG00000107104 60.2208 1312.7390

ENSG00000121552 20.6279 407.8878

ENSG00000147403 598.9158 24064.5500

ENSG00000187045 1.4280 2.6281

ENSG00000145506 1.9193 2.1102

ENSG00000179772 1.1826 1.5513

ENSG00000132478 75.0839 515.6123

ENSG00000047662 20.1278 54.8762

ENSG00000105509 .8296 2.8659

ENSG00000042753 72.8333 767.0391

ENSG00000160746 11.7494 50.0891

ENSG00000079102 2.9181 11.4993

ENSG00000197321 46.6464 823.5935

ENSG00000171459 .0041 .0001

ENSG00000167759 .0858 .0259

ENSG00000236446 .0129 .0003

ENSG00000176884 .3995 .0994

ENSG00000172985 9.3651 173.2715

ENSG00000100926 133.1042 365.4355

ENSG00000139865 1.4536 2.7363

ENSG00000172578 361.5083 105131.6000

ENSG00000149930 87.7541 521.9744

ENSG00000058866 10.0820 92.2855

ENSG00000161243 1.7779 1.8562

ENSG00000166886 33.8996 298.4417

ENSG00000041982 151.3378 21889.9100

ENSG00000184009 3933.3370 1854764.0000

ENSG00000112576 241.2962 10481.7700

ENSG00000183624 311.2862 74835.7300

ENSG00000185958 1.0910 .4251

ENSG00000198053 33.0333 849.5690

ENSG00000006432 19.5219 86.1520

ENSG00000163512 55.1973 234.9642

ENSG00000143847 2.2470 1.8054

ENSG00000149571 3.6159 5.2440

ENSG00000119913 .0540 .0014

ENSG00000136842 23.7866 467.8823

ENSG00000100065 3.8387 14.9317

ENSG00000186971 .0032 .0001

ENSG00000162390 3.5803 10.1636

ENSG00000113594 32.0642 2373.8650

ENSG00000138081 355.3750 10979.2900

ENSG00000156508 4398.4080 1531551.0000

ENSG00000165995 13.4754 67.2179

ENSG00000154358 35.7516 529.3134

ENSG00000188152 2.2312 1.0166

ENSG00000171045 34.7689 193.9197

ENSG00000132155 188.3726 1044.5860

ENSG00000167548 224.0645 10668.2800

ENSG00000160691 64.9320 187.0999

ENSG00000107249 5.5158 28.3087

ENSG00000150907 123.7039 2867.0740

ENSG00000006283 .9840 .5488

ENSG00000180921 6.0212 14.2004

ENSG00000112079 100.0318 804.4680

ENSG00000167971 14.4827 26.8271

ENSG00000076356 5.9108 19.8241

ENSG00000151332 41.2309 78.2655

ENSG00000185420 42.9811 326.6074

ENSG00000007047 121.3242 409.4240

ENSG00000107186 11.6573 194.2877

ENSG00000103855 12.5747 143.0560

ENSG00000135535 287.8227 8796.5710

ENSG00000177575 94.3298 48079.3400

ENSG00000160190 67.5552 461.1425

ENSG00000120053 40.1515 100.0049

ENSG00000181143 27.9935 7685.8750

ENSG00000167487 6.3282 4.6111

ENSG00000148396 147.9118 1417.2100

ENSG00000147140 372.4461 8446.4430

ENSG00000061938 174.7687 8503.9660

ENSG00000176896 9.0175 14.3521

ENSG00000156414 2.0600 1.7240

ENSG00000137962 14.5609 213.8618

ENSG00000204033 .0301 .0005

ENSG00000176945 3.5726 14.8175

ENSG00000197128 8.9567 29.6907

ENSG00000175497 4.3443 8.5166

ENSG00000108679 81.5835 7494.2900

ENSG00000183798 .5727 .2395

ENSG00000196535 202.9007 8314.9000

ENSG00000187672 3.2721 9.3322

ENSG00000004534 452.7556 44463.2100

ENSG00000133119 131.4618 9061.9350

ENSG00000158022 .1131 .0150

ENSG00000254245 75.1837 2216.2540

ENSG00000197969 133.9284 1638.3130

ENSG00000010030 7.8154 118.1111

ENSG00000143632 .6917 .0995

ENSG00000116213 50.8749 70.2723

ENSG00000038427 28.8475 1598.0350

ENSG00000135476 99.5710 5403.8300

ENSG00000054118 201.9936 2076.7950

ENSG00000186583 1.6688 3.4878

ENSG00000227507 215.2408 15778.7300

ENSG00000251322 17.8770 535.9474

ENSG00000135119 20.9345 155.2370

ENSG00000120733 170.8516 1281.5960

ENSG00000183914 1.5259 3.2522

ENSG00000101680 3.3507 2.2753

ENSG00000135226 1.5469 12.9539

ENSG00000155657 118.0626 8335.0550

ENSG00000157766 1.1324 2.4278

ENSG00000197566 6.8283 5.4179

ENSG00000007968 85.1658 3683.8290

ENSG00000072135 267.6821 17786.0500

ENSG00000099377 6.4374 24.6533

ENSG00000145555 7.7501 27.3262

ENSG00000187764 152.9820 3848.7310

ENSG00000186367 .0368 .0014

ENSG00000091317 114.7126 2511.7090

ENSG00000143469 .7193 .5748

ENSG00000114739 7.4116 33.8364

ENSG00000137642 414.1139 61369.3600

ENSG00000141012 30.8326 291.2036

ENSG00000163531 10.0051 134.4623

ENSG00000060688 52.1947 289.5193

ENSG00000157657 6.8846 21.3943

ENSG00000135069 22.9945 349.5981

ENSG00000129646 6.3374 15.5644

ENSG00000171408 6.9982 33.2534

ENSG00000101577 77.1493 870.3483

ENSG00000188738 2.4049 8.0070

**Supplementary Table S3. Correlation between studied mutable motifs and the sequence context of somatic mutations (G:C sites) in various cancer types.**

-----------------------------------------------------------------

Tissue #Mut. Pol eta (C:G sites) Pol theta (C:G sites)

Ratio t-test MC_P Ratio t-test MC_P

-----------------------------------------------------------------

Bladder 23127 .994 NSE .948 NSE

Blood 6331 1.056* 21.378 <0.001 1.111* 37.231 <0.001

Brain 23354 1.081* 51.872 <0.001 1.153* 87.094 <0.001

Breast 10327 1.017* 6.695 <0.001 1.007 NSE

Cervix 23034 .987 NSE .933 NSE

Colon 132513 1.078* 118.944 <0.001 1.149* 198.393 <0.001

Kidney 16690 1.016* 10.940 <0.001 1.042* 25.286 <0.001

Liver 42221 1.019* 18.826 <0.001 1.056* 51.316 <0.001

Lung 58116 1.003* 5.651 <0.001 1.017* 23.591 <0.001

Ovary 10973 1.030* 16.612 <0.001 1.066* 32.798 <0.001

Pancreas 25544 1.070* 47.517 <0.001 1.130* 77.401 <0.001

Prostate 9506 1.050* 23.745 <0.001 1.122* 51.693 <0.001

Rectum 20315 1.056* 34.965 <0.001 1.130* 71.003 <0.001

Skin 262554 .881 NSE .978 NSE

Stomach 92882 1.093* 115.544 <0.001 1.163* 177.625 <0.001

Uterus 41875 1.073* 63.750 <0.001 1.137* 101.617 <0.001

------------------------------------------------------------------

NSE (no significant excess) indicates the absence of a significant excess of mutations in mutable motifs suggesting there to be no association between mutagenesis and DNA sequence motifs. The significance of any excess was measured by means of the Student *t* and Monte Carlo (MC) tests. The asterisk (*) denotes that the corresponding P < 0.002 (critical value = 3.1); this is a conservative estimate of the critical overall value of the *t*-test having allowed for multiple testing by means of the Bonferroni correction (4 x 6 = 24). “Ratio” is the mean weight of mutated sites divided by the mean weight of non-mutated sites.

**Supplementary Table S4. Correlation between studied mutable motifs and the sequence context of somatic mutations (A:T sites) in various cancer types.**

-------------------------------------------------------------

Tissue #Mut. Pol eta (A:T sites) Pol theta (A:T sites)

Ratio t-test MC_P Ratio t-test MC_P

-------------------------------------------------------------

Bladder 23127 1.007 NSE .955 NSE

Blood 6331 1.006 NSE .989 NSE

Brain 23354 1.008* 2.926 0.004 .987 NSE

Breast 10327 1.018* 8.920 <0.001 .987 NSE

Cervix 23034 1.014* 2.984 0.001 1.028* 5.846 <0.001

Colon 132513 1.008* 6.355 <0.001 1.000 NSE

Kidney 16690 1.008* 3.971 <0.001 .996 NSE

Liver 42221 1.000 NSE .972 NSE

Lung 58116 .965 NSE .942 NSE

Ovary 10973 1.008* 2.602 0.008 .992 NSE

Pancreas 25544 1.011* 4.050 <0.001 .994 NSE

Prostate 9506 1.018* 4.549 <0.001 .983 NSE

Rectum 20315 1.023* 7.849 <0.001 1.032* 11.011 <0.001

Skin 262554 1.016* 10.094 <0.001 1.055* 33.706 <0.001

Stomach 92882 .995 NSE 1.003* 3.059 <0.001

Uterus 41875 1.017* 7.110 <0.001 .989 NSE

-------------------------------------------------------------

NSE (no significant excess) indicates the absence of a significant excess of mutations in mutable motifs suggesting there to be no association between mutagenesis and DNA sequence motifs. The significance of any excess was measured by means of the Student *t* and Monte Carlo (MC) tests. The asterisk (*) denotes that the corresponding P < 0.002 (critical value = 3.1); this is a conservative estimate of the critical overall value of the *t*-test having allowed for multiple testing by means of the Bonferroni correction (4 x 6 = 24). “Ratio” is the mean weight of mutated sites divided by the mean weight of non-mutated sites.

**Supplementary Table S5. Control experiments using shuffled weight matrices.**

-----------------------------------------------------------------------

Tissue %errors G:C %errors A:T

----------------------------- --------------------

AID Pol eta Pol theta Pol eta Pol theta

Bladder - 1. 1. - -

# Blood - - - - -

**Blood:**

**GCB lymphomas - - 0.01 - -**

**Blood:**

**MALY-DE lymphomas - - - - 0.02**

# Brain 0.03 0.15 0.07 - 0.20

Breast - 0.92 0.70 - 0.75

Cervix - 0.93 1. - -

Colon 0.35 0.04 0.09 - 1.00

**Kidney** - 0.04 - - -

# Liver - - - - -

Lung - 0.85 0.96 - -

# Ovary - - - - -

**Pancreas**  **- 0.14 0.11 - -**

Prostate - 0.30 0.22 - -

Rectum 0.21 - - 1.00 -

**Skin** **- 0.05 - - -**

Stomach 0.20 0.15 0.04 1.00 0.99

Uterus - 0.06 0.13 1.00 1.00

-----------------------------------------------------------------------

Tissues with the maximum rate of errors less than or equal to 0.2 are assumed to be reliable and can be used for further analysis.

**Supplementary Table S6. Functional annotation of driver and non-driver genes (the David website, https://david.ncifcrf.gov/).**

| David feature Feature name P (after Bonferroni  class correction) |
| --- |
| **Driver genes** |
| UP_SEQ_FEATURE Domain:Ras-GEF 9.9E-4 |
| UP_KEYWORDS Phosphoprotein 3.8E-5 |
| UP_KEYWORDS Ubl conjugation 1.9E-4 |
| UP_KEYWORDS Glycoprotein 2.1E-3 |
| UP_KEYWORDS Methylation 5.9E-3 |
| UP_KEYWORDS Disease mutation 9.7E-3 |
| UP_KEYWORDS Isopeptide bond 1.6E-2 |
| UP_KEYWORDS Polymorphism 1.6E-2 |
| GOTERM_CC_DIRECT Plasma membrane 9.3E-3 |
| GOTERM_CC_DIRECT Nuclear chromatin 4.6E-2 |
| KEGG_PATHWAY Thyroid hormone 2.0E-6  signaling pathway |
| KEGG_PATHWAY Colorectal cancer 2.5E-3 |
| KEGG_PATHWAY Proteoglycans in 7.0E-3  cancer |
| KEGG_PATHWAY Viral 7.0E-3  carcinogenesis |
| KEGG_PATHWAY Prostate cancer 7.1E-3 |
| KEGG_PATHWAY Hepatitis B 1.5E-2 |
| KEGG_PATHWAY HTLV-I infection 1.9E-2 |
| KEGG_PATHWAY Rap1 signaling 2.1E-2  pathway |
| KEGG_PATHWAY Epstein-Barr 2.3E-2  virus infection |
| KEGG_PATHWAY Pathways in cancer 2.5E-2 |
| KEGG_PATHWAY Endometrial cancer 2.5E-2 |
| KEGG_PATHWAY Apoptosis 4.4E-2 |
| KEGG_PATHWAY Central carbon 4.5E-2  metabolism in cancer |
| BIOCARTA CTCF 7.7E-3 |
| BIOCARTA Tumor Suppressor 1.5E-2  Arf Inhibits  Ribosomal Biogenesis |
| BIOCARTA Phospholipids as 5.1E-2  signaling intermediaries |
| INTERPRO Ras guanine 4.4E-4  nucleotide exchange  factor, domain |
| INTERPRO Guanine-nucleotide 4.4E-4  dissociation stimulator  CDC25 |
| SMART RasGEF 8.6E-4 |
| SMART RasGEFN 4.7E-2 |
| **Non-driver genes** |
| UP_SEQ_FEATURE Splice variant 6.0E-3 |
| UP_SEQ_FEATURE Domain:Fibronectin 2.2E-2  type-III 3 |
| UP_SEQ_FEATURE sequence variant 2.4E-2 |
| UP_KEYWORDS Alternative splicing 1.2E-6 |
| UP_KEYWORDS Polymorphism 1.7E-4 |
| UP_KEYWORDS Phosphoprotein 3.0E-3 |
| UP_KEYWORDS Immunoglobulin 4.8E-2  domain |

**Supplementary Table S7. Correlation between studied mutable motifs and the sequence context of somatic mutations in MALY-DE patients.**

-------------------------------------

Ratio t-test MC_P

value

-------------------------------------

**Patient = DO27787**

G/C N=7114

AID 1.052* 12.055 <0.001

POL ETA 1.039* 12.462 <0.001

POL THETA 1.066* 19.574 <0.001

A/T N=8233

POL ETA 1.033* 12.491 <0.001

POL THETA 0.973 NSE 1.000

**Patient = DO27795**

G/C N=4356

AID 1.069* 12.255 <0.001

POL ETA 1.043* 10.992 <0.001

POL THETA 1.095* 21.408 <0.001

A/T N=3432

POL ETA 1.015* 3.627 <0.001

POL THETA 0.971 NSE 1.000

**Patient = DO27797**

G/C N=8949

AID 1.111* 28.728 <0.001

POL ETA 1.061* 20.862 <0.001

POL THETA 1.105* 35.159 <0.001

A/T N=6623

POL ETA 1.025* 8.684 <0.001

POL THETA 0.994 NSE 0.969

Patient = DO27799

G/C N=4076

AID 1.126* 22.305 <0.001

POL ETA 1.062* 14.911 <0.001

POL THETA 1.119* 25.561 <0.001

A/T N=4902

POL ETA 0.990 NSE 0.997

POL THETA 1.042* 11.400 <0.001

Patient = DO27801

G/C N=10820

AID 1.124* 35.761 <0.001

POL ETA 1.056* 22.171 <0.001

POL THETA 1.095* 34.163 <0.001

A/T N=13903

POL ETA 1.028* 13.465 <0.001

POL THETA 0.978 NSE 1.000

Patient = DO27803

G/C N=12712

AID 1.075* 22.706 <0.001

POL ETA 1.050* 20.725 <0.001

POL THETA 1.088* 34.718 <0.001

A/T N=17968

POL ETA 1.028* 15.117 <0.001

POL THETA 0.973 NSE 1.000

Patient = DO27805

G/C N=10553

AID 1.091* 25.659 <0.001

POL ETA 1.063* 23.010 <0.001

POL THETA 1.099* 35.663 <0.001

A/T N=10002

POL ETA 1.025* 10.199 <0.001

POL THETA 0.995 NSE 0.985

Patient = DO27809

G/C N=13700

AID 1.094* 30.548 <0.001

POL ETA 1.039* 17.017 <0.001

POL THETA 1.089* 36.791 <0.001

A/T N=11092

POL ETA 1.021* 8.994 <0.001

POL THETA 0.987 NSE 1.000

Patient = DO27815

G/C N=3539

AID 1.072* 12.140 <0.001

POL ETA 1.056* 11.710 <0.001

POL THETA 1.103* 19.247 <0.001

A/T N=2436

POL ETA 1.001 NSE 0.457

POL THETA 1.000 NSE 0.502

Patient = DO27819

G/C N=5784

AID 1.086* 18.297 <0.001

POL ETA 1.031* 8.547 <0.001

POL THETA 1.080* 21.729 <0.001

A/T N=6166

POL ETA 1.021* 6.259 <0.001

POL THETA 0.986 NSE 1.000

Patient = DO27764

G:C N=4947

AID 1.099* 18.995 <0.001

POL ETA 1.062* 16.095 <0.001

POL THETA 1.102* 25.195 <0.001

A:T N=3401

POL ETA 1.021* 5.141 <0.001

POL THETA 0.971 NSE 1.000

Patient = DO27821

G/C N=6325

AID 1.112* 24.864 <0.001

POL ETA 1.048* 14.247 <0.001

POL THETA 1.122* 34.180 <0.001

A/T N=6316

POL ETA 1.019* 5.743 <0.001

POL THETA 0.984 NSE 1.000

Patient = DO27823

G/C N=3207

AID 1.028* 4.218 <0.001

POL ETA 1.075* 14.879 <0.001

POL THETA 1.123* 23.561 <0.001

A/T N=2017

POL ETA 0.971 NSE 1.000

POL THETA 0.969 NSE 1.000

Patient = DO27825

G/C N=4168

AID 1.098* 17.784 <0.001

POL ETA 1.057* 14.698 <0.001

POL THETA 1.121* 27.175 <0.001

A/T N=2154

POL ETA 0.990 NSE 0.966

POL THETA 0.974 NSE 1.000

Patient = DO27765

G/C N=3690

AID 1.098* 17.588 <0.001

POL ETA 1.073* 16.213 <0.001

POL THETA 1.109* 22.934 <0.001

A/T N=2609

POL ETA 0.994 NSE 0.882

POL THETA 0.999 NSE 0.563

Patient = DO27767

G/C N=7358

AID 1.077* 18.468 <0.001

POL ETA 1.059* 18.537 <0.001

POL THETA 1.095* 27.462 <0.001

A/T N=8130

POL ETA 1.006 NSE <0.001

POL THETA 1.026* 8.130 <0.001

Patient = DO27769

G/G N=6662

AID 1.116* 26.999 <0.001

POL ETA 1.037* 11.117 <0.001

POL THETA 1.079* 21.734 <0.001

A/T N=5445

POL ETA 1.016* 4.695 <0.001

POL THETA 0.997 NSE 0.779

Patient = DO27773

G/C N=6337

AID 1.066* 14.407 <0.001

POL ETA 1.055* 15.471 <0.001

POL THETA 1.116* 31.726 <0.001

A/T N=4871

POL ETA 0.999 NSE 0.571

POL THETA 0.997 NSE .841

Patient = DO27775

G/C N=4421

AID 1.139* 24.621 <0.001

POL ETA 1.056* 14.373 <0.001

POL THETA 1.105* 24.721 <0.001

A/T N=3157

POL ETA 1.004 NSE 0.161

POL THETA 1.004 NSE 0.180

Patient = DO27781

G/C N=6943

AID 1.095* 22.506 <0.001

POL ETA 1.050* 14.899 <0.001

POL THETA 1.093* 27.020 <0.001

A/T N=6274

POL ETA 1.012* 3.658 <0.001

POL THETA 0.977 NSE 1.000

Patient = DO27785

G/C N=6147

AID 1.113* 25.460 <0.001

POL ETA 1.043* 12.230 <0.001

POL THETA 1.107* 29.754 <0.001

A/T N=6744

POL ETA 1.013* 4.429 <0.001

POL THETA 0.984 NSE 1.000

Patient = DO27763

G/C N=3960

AID 1.085* 14.376 <0.001

POL ETA 1.041* 9.381 <0.001

POL THETA 1.105* 21.713 <0.001

A/T N=1900

POL ETA 1.059* 10.163 <0.001

POL THETA 0.970 NSE 1.000

**----------------------------------------------**

NSE (no significant excess) indicates the absence of a significant excess of mutations in mutable motifs suggesting there to be no association between mutagenesis and DNA sequence motifs. The significance of any excess was measured by means of the Student *t* and Monte Carlo (MC) tests. The asterisk (*) denotes that the corresponding P < 0.002 (critical value = 3.1); this is a conservative estimate of the critical overall value of the *t*-test having allowed for multiple testing by means of the Bonferroni correction (4 x 6 = 24). “Ratio” is the mean weight of mutated sites divided by the mean weight of non-mutated sites.

**Supplementary Table S8. Analysis of mRNA expression (FPKM values) in sets of driver and non-driver genes.**

| **Group of genes** | **Mean value** | **Variance** |
| --- | --- | --- |
| Driver | 146 | 68680 |
| Non-driver | 124 | 47247 |

The difference between mean values is not statistically significant (t-test P value = 0.086).

The difference between variance values is statistically significant (F-test P value =0.007).

**Supplementary Figure S1. Number of nucleotides that were used for the construction of weight matrices for DNA pol η (A - G:C sites; B - A:T sites) and pol θ (C - G:C sites; D - A:T sites). Position 0 is not used in calculations.**

(A)

-5 -4 -3 -2 -1 0 +1 +2 +3 +4 +5

A 50 58 58 35 52 0 42 32 56 58 59

T 59 73 39 70 55 0 58 52 86 56 41

G 47 54 52 43 41 224 28 58 49 70 60

C 68 39 75 76 76 0 96 82 33 40 64

(B)

-5 -4 -3 -2 -1 0 +1 +2 +3 +4 +5

A 108 121 108 80 125 388 111 63 122 103 87

T 91 65 54 113 107 0 131 101 85 67 89

G 70 91 116 65 83 0 41 101 89 72 104

C 119 111 110 130 73 0 105 123 92 146 108

(C)

-5 -4 -3 -2 -1 0 +1 +2 +3 +4 +5

A 16 23 15 19 7 0 11 8 23 16 12

T 14 15 25 24 8 0 14 26 16 18 12

G 19 21 20 20 14 69 15 15 11 18 22

C 20 10 9 6 40 0 29 20 19 17 23

(D)

-5 -4 -3 -2 -1 0 +1 +2 +3 +4 +5

A 16 40 29 33 65 139 35 17 21 22 30

T 40 27 26 43 12 0 38 35 44 39 25

G 43 49 63 41 31 0 36 42 36 45 48

C 40 23 21 22 31 0 30 45 38 33 36

**Supplementary Figure S2. Set of mutations produced by pol η *in vitro*. These mutations were generated by classic gap-filling DNA synthesis (T Matsuda, K Bebenek, C Masutani, I B Rogozin, F Hanaoka, T A Kunkel, Error rate and specificity of human and murine DNA polymerase eta J Mol Biol. 2001, 312:335-46).**

1,C->T,1

2,A->T,1

3,G->A,1

4,C->T,2

5,T->C,7

7,T->C,4

8,T->C,5

9,G->T,1

9,G->A,3

10,C->T,1

12,C->T,1

13,G->T,2

13,G->A,4

13,G->C,1

14,T->A,2

14,T->C,3

15,C->T,6

16,T->C,3

17,C->T,2

17,C->A,1

18,A->C,1

19,C->G,1

19,C->T,3

20,T->A,2

20,T->C,6

20,T->G,1

21,G->A,2

22,G->A,3

22,G->C,4

23,T->A,1

23,T->C,1

23,T->G,1

25,A->C,1

25,A->G,1

26,A->C,1

27,A->C,2

27,A->G,3

27,A->T,3

28,A->C,1

28,A->T,1

30,A->G,2

30,A->T,2

31,A->C,1

31,A->G,1

33,A->C,1

33,A->T,4

34,A->C,2

34,A->G,1

34,A->T,1

36,C->A,2

37,A->C,1

37,A->G,2

37,A->T,3

38,C->G,1

39,C->A,1

41,T->C,2

42,G->A,1

42,G->C,1

43,G->A,1

45,G->T,3

45,G->A,3

46,C->T,2

47,C->A,2

48,C->T,2

49,A->C,1

49,A->T,3

50,A->T,1

51,T->A,1

51,T->C,12

51,T->G,3

52,A->C,1

52,A->G,1

53,C->T,1

55,C->T,1

57,A->G,2

58,A->C,2

58,A->G,2

58,A->T,1

60,C->T,1

61,G->A,5

61,G->C,1

62,C->G,1

63,C->T,1

64,T->C,4

64,T->G,2

65,C->G,1

65,C->T,6

65,C->A,2

66,T->A,2

66,T->C,4

66,T->G,1

69,C->A,2

71,G->A,1

73,G->C,1

75,G->A,2

76,T->A,1

77,T->C,3

80,C->T,1

81,C->T,2

82,G->T,1

83,A->G,1

83,A->T,5

84,T->C,11

84,T->G,2

85,T->C,6

85,T->G,1

86,C->A,1

87,A->T,2

88,T->C,16

89,T->A,1

89,T->C,8

89,T->G,2

90,A->C,2

90,A->G,1

90,A->T,1

91,A->T,5

92,T->C,4

93,G->A,2

94,C->T,1

94,C->A,1

95,A->T,1

96,G->T,1

96,G->A,5

96,G->C,2

97,C->T,1

98,T->C,4

99,G->A,2

101,C->A,2

102,A->C,1

102,A->T,1

103,C->T,1

104,G->A,2

105,A->G,3

105,A->T,1

106,C->T,1

107,A->T,1

108,G->T,1

109,G->A,1

110,T->C,8

111,T->A,1

111,T->C,12

112,T->C,7

112,T->G,1

116,G->T,2

116,G->A,5

116,G->C,1

117,A->C,3

117,A->G,3

118,C->T,1

119,T->A,2

119,T->C,6

120,G->A,1

120,G->C,2

121,G->T,1

121,G->A,1

122,A->C,1

122,A->G,1

122,A->T,1

125,G->A,1

127,G->A,1

128,G->T,1

131,A->G,1

132,G->A,1

132,G->C,3

133,T->C,1

134,G->A,1

135,A->G,1

136,G->T,1

136,G->A,1

136,G->C,1

137,C->T,1

138,G->C,1

140,A->G,1

140,A->T,2

141,A->G,2

141,A->T,1

142,C->G,1

142,C->T,1

143,G->T,1

143,G->C,1

144,C->A,1

145,A->T,1

146,A->C,1

146,A->G,1

146,A->T,3

147,T->C,10

148,T->A,1

148,T->C,16

149,A->C,1

149,A->G,2

149,A->T,4

150,A->C,1

150,A->G,3

150,A->T,4

151,T->A,1

151,T->C,2

152,G->A,1

152,G->C,4

153,T->A,1

153,T->C,2

154,G->A,1

155,A->C,3

155,A->T,1

156,G->C,3

157,T->C,12

158,T->C,12

159,A->C,3

159,A->T,2

160,G->T,2

160,G->A,3

161,C->T,3

162,T->C,3

162,T->G,1

163,C->T,4

163,C->A,2

164,A->C,2

164,A->G,2

166,T->C,5

167,C->T,2

168,A->C,2

168,A->T,2

169,T->A,1

169,T->C,6

170,T->A,1

170,T->C,7

170,T->G,1

171,A->C,3

172,G->A,2

173,G->C,1

174,C->T,1

175,A->G,2

178,C->A,1

179,C->T,1

180,A->C,1

180,A->T,1

181,G->A,2

182,G->T,1

182,G->A,2

182,G->C,2

183,C->T,1

184,T->A,1

184,T->C,10

184,T->G,1

185,T->A,2

185,T->C,9

185,T->G,1

186,T->A,1

186,T->C,11

186,T->G,1

187,A->C,3

187,A->G,3

188,C->T,1

188,C->A,1

189,A->C,1

190,C->T,2

191,T->A,1

191,T->C,15

192,T->A,2

192,T->C,12

192,T->G,1

193,T->C,9

193,T->G,2

194,A->C,3

194,A->T,5

195,T->C,5

196,G->A,2

197,C->T,1

198,T->C,6

198,T->G,1

199,T->C,4

199,T->G,1

200,C->G,1

202,G->T,1

202,G->A,2

203,G->A,1

204,C->T,3

205,T->C,7

205,T->G,2

206,C->T,1

207,G->A,2

208,T->C,14

208,T->G,1

209,A->C,4

209,A->T,7

210,T->C,9

211,G->A,1

212,T->A,1

212,T->C,19

213,T->C,1

214,G->A,3

215,T->C,2

215,T->G,1

216,G->T,1

216,G->A,5

217,T->A,1

217,T->C,3

217,T->G,1

219,G->T,1

219,G->A,3

220,A->C,2

220,A->G,1

220,A->T,2

221,A->C,1

221,A->G,1

221,A->T,1

222,T->A,1

222,T->C,15

222,T->G,1

223,T->A,2

223,T->C,2

224,G->A,4

225,T->C,1

227,A->C,1

227,A->T,3

228,G->A,3

229,C->T,2

230,G->T,1

230,G->A,1

232,A->C,1

232,A->T,1

233,T->A,1

233,T->C,14

233,T->G,1

234,A->C,10

234,A->T,5

235,A->C,1

235,A->G,2

236,C->T,1

236,C->A,1

237,A->C,1

237,A->T,2

238,A->C,3

238,A->G,1

238,A->T,5

239,T->A,1

239,T->C,11

240,T->A,2

240,T->C,16

241,T->A,1

241,T->C,7

241,T->G,1

242,C->T,3

242,C->A,1

243,A->G,1

247,A->C,1

247,A->T,2

248,G->T,1

250,A->C,1

250,A->G,2

250,A->T,5

251,A->C,3

251,A->T,3

252,A->C,2

252,A->G,1

252,A->T,1

253,C->T,1

254,A->C,1

254,A->T,2

255,G->A,2

255,G->C,1

256,C->T,1

257,T->A,2

257,T->C,9

257,T->G,1

258,A->C,9

258,A->G,2

258,A->T,9

259,T->C,1

260,G->A,2

261,A->C,1

261,A->G,4

262,C->G,1

263,C->T,2

263,C->A,1

264,A->C,1

264,A->G,1

264,A->T,2

265,T->A,1

265,T->C,13

266,G->A,2

266,G->C,1

267,A->C,3

267,A->T,4

268,T->C,7

268,T->G,1

269,T->A,4

269,T->C,16

270,A->C,1

270,A->G,1

272,G->A,4

273,A->C,1

273,A->G,1

274,A->C,2

275,T->C,17

276,T->C,4

277,C->T,5

277,C->A,1

278,A->G,1

279,C->G,2

279,C->T,2

280,T->C,2

281,G->C,1

282,G->T,1

282,G->A,2

282,G->C,1

283,C->T,1

284,C->T,1

285,G->T,1

285,G->A,3

285,G->C,1

286,T->C,4

286,T->G,2

288,G->A,3

289,T->A,3

289,T->C,18

290,T->A,1

290,T->C,9

291,T->C,6

292,T->A,1

292,T->C,14

293,A->G,2

295,A->G,1

296,A->G,12

298,G->C,1

299,T->A,2

299,T->C,11

300,C->T,1

301,G->C,1

302,T->A,2

302,T->C,2

303,G->T,1

303,G->A,2

303,G->C,6

304,A->G,2

304,A->T,2

305,C->T,1

306,T->A,1

306,T->C,1

306,T->G,1

308,G->T,1

308,G->A,2

309,G->A,1

310,A->T,2

311,A->T,1

312,A->C,2

312,A->T,1

313,A->G,1

313,A->T,1

314,C->T,1

315,C->G,1

315,C->T,1

315,C->A,1

317,T->C,5

319,G->T,2

319,G->A,2

319,G->C,1

320,C->T,1

320,C->A,1

321,G->A,1

322,T->C,4

323,T->A,1

323,T->C,16

323,T->G,1

324,A->G,1

324,A->T,3

326,C->A,1

328,A->C,2

328,A->T,2

329,A->C,1

329,A->G,6

330,C->T,1

331,T->C,11

332,T->C,4

332,T->G,1

333,A->C,1

333,A->T,3

334,A->C,4

334,A->T,4

335,T->C,4

335,T->G,1

336,C->T,2

336,C->A,1

337,G->T,4

337,G->A,5

338,C->T,1

340,T->A,2

340,T->C,17

341,T->C,5

343,C->T,1

343,C->A,2

344,A->G,1

345,G->T,1

345,G->A,1

346,C->T,5

347,A->C,1

349,A->C,3

349,A->T,7

350,T->A,1

350,T->C,2

351,C->T,1

351,C->A,3

353,C->G,2

354,C->T,1

356,T->A,2

356,T->C,4

357,T->C,7

357,T->G,1

358,T->C,2

359,C->T,1

360,G->A,2

361,C->A,1

363,A->C,1

363,A->G,1

363,A->T,3

364,G->A,3

365,C->A,1

366,T->C,3

367,G->A,2

368,G->T,2

368,G->A,2

370,G->A,2

371,T->C,5

371,T->G,2

372,A->G,1

372,A->T,3

373,A->C,1

373,A->G,1

373,A->T,2

374,T->C,8

374,T->G,1

376,G->T,2

376,G->A,1

376,G->C,1

379,A->C,2

379,A->G,1

379,A->T,2

380,A->G,1

381,G->A,1

381,G->C,1

382,A->T,2

384,G->A,1

385,C->A,1

386,C->T,2

387,C->T,1

389,C->A,1

390,A->C,1

390,A->G,1

392,C->T,1

393,G->A,1

393,G->C,1

394,A->C,3

394,A->G,4

394,A->T,5

395,T->A,2

395,T->C,4

395,T->G,1

396,C->T,4

396,C->A,1

397,G->A,1

399,C->T,1

401,T->A,1

401,T->C,12

402,T->A,1

402,T->C,4

402,T->G,3

403,C->T,1

404,C->T,1

404,C->A,1

406,A->T,2

407,A->G,6

407,A->T,3

408,C->G,1

408,C->T,1

409,A->G,1

409,A->T,1

410,G->T,2

410,G->A,2

410,G->C,1

Sequence:lacZa gene -220 - +200

CAGCTGTTGCCCGTCTCACTGGTGAAAAGAAAAACCACCCTGGCGCCCAATACGCAAACC

GCCTCTCCCCGCGCGTTGGCCGATTCATTAATGCAGCTGGCACGACAGGTTTCCCGACTG

GAAAGCGGGCAGTGAGCGCAACGCAATTAATGTGAGTTAGCTCACTCATTAGGCACCCCA

GGCTTTACACTTTATGCTTCCGGCTCGTATGTTGTGTGGAATTGTGAGCGGATAACAATT

TCACACAGGAAACAGCTATGACCATGATTACGAATTCACTGGCCGTCGTTTTACAACGTC

GTGACTGGGAAAACCCTGGCGTTACCCAACTTAATCGCCTTGCAGCACATCCCCCTTTCG

CCAGCTGGCGTAATAGCGAAGAGGCCCGCACCGATCGCCCTTCCCAACAGCTGCGCA

**Supplementary Figure S3. Set of mutations produced by pol** **θ *in vitro*. These mutations were generated by classic gap-filling DNA synthesis (Arana ME, Seki M, Wood RD, Rogozin IB, Kunkel TA. Low-fidelity DNA synthesis by human DNA polymerase theta. Nucleic Acids Res. 2008, 36:3847-56).**

294,A->C,1,

313,T->A,1,

313,T->A,1,

256,A->T,1,

331,T->C,1,

66,T->C,1,

271,T->G,1,

347,T->C,1,

313,T->A,1,

312,T->C,1,

321,T->C,1,

92,T->C,1,

48,T->C,1,

70,T->C,1,

186,C->c,1,

317,C->T,1,

254,G->A,1,

222,T->C,1,

313,T->A,1,

302,G->T,1,

303,T->G,1,

331,T->A,1,

371,A->G,1,

375,A->G,1,

377,C->T,1,

94,T->C,1,

142,G->A,1,

295,C->G,1,

91,G->T,1,

129,T->A,1,

303,T->C,1,

377,C->T,1,

373,C->T,1,

294,A->C,1,

243,A->C,1,

247,T->C,1,

291,G->A,1,

188,C->T,1,

63,C->c,1,

350,C->c,1,

341,G->C,1,

201,G->A,1,

177,T->C,1,

221,T->A,1,

280,G->A,1,

48,T->C,1,

195,T->C,1,

292,A->T,1,

337,T->C,1,

378,G->C,1,

17,C->A,1,

19,A->C,1,

70,T->A,1,

152,T->C,1,

371,A->G,1,

209,A->G,1,

306,C->T,1,

358,C->T,1,

177,T->C,1,

178,G->C,1,

322,T->C,1,

337,T->C,1,

220,A->T,1,

250,T->C,1,

181,T->C,1,

119,C->T,1,

26,C->T,1,

146,A->G,1,

220,A->T,1,

303,T->C,1,

377,C->T,1,

386,C->A,1,

107,G->C,1,

221,T->A,1,

144,T->C,1,

221,T->A,1,

119,C->T,1,

188,C->T,1,

331,T->C,1,

167,T->A,1,

288,T->C,1,

166,T->C,1,

271,T->G,1,

220,A->T,1,

140,T->C,1,

286,A->G,1,

193,G->A,1,

67,T->C,1,

26,C->T,1,

54,C->T,1,

61,G->A,1,

220,A->T,1,

157,A->G,1,

264,G->A,1,

303,T->C,1,

383,T->c,1,

220,A->T,1,

138,G->T,1,

247,T->C,1,

331,T->C,1,

91,G->T,1,

71,T->A,1,

350,C->T,1,

378,G->C,1,

358,C->T,1,

294,A->C,1,

383,T->C,1,

166,T->C,1,

174,T->C,1,

209,A->G,1,

382,T->C,1,

63,C->T,1,

94,T->C,1,

368,C->T,1,

298,T->G,1,

313,T->A,1,

94,T->C,1,

288,T->C,1,

304,T->C,1,

330,A->C,1,

48,T->C,1,

106,A->G,1,

195,T->G,1,

205,T->C,1,

331,T->C,1,

184,G->A,1,

243,A->C,1,

135,T->C,1,

129,T->A,1,

292,A->G,1,

166,T->C,1,

183,C->T,1,

386,C->A,1,

383,T->C,1,

195,T->C,1,

278,A->G,1,

326,G->A,1,

92,T->C,1,

93,T->A,1,

56,C->T,1,

257,T->A,1,

130,T->A,1,

180,T->C,1,

231,G->A,1,

273,T->C,1,

92,T->C,1,

345,G->A,1,

71,T->A,1,

26,C->T,1,

70,T->A,1,

171,A->G,1,

48,T->C,1,

97,C->T,1,

102,G->A,1,

80,T->C,1,

318,G->C,1,

48,T->C,1,

256,A->T,1,

279,C->T,1,

63,C->T,1,

1,C->T,1,

63,C->G,1,

347,T->C,1,

358,C->T,1,

365,G->A,1,

97,C->T,1,

129,T->A,1,

70,T->A,1,

331,T->A,1,

347,T->C,1,

114,G->A,1,

205,T->C,1,

334,C->T,1,

92,T->C,1,

173,T->C,1,

91,G->T,1,

194,T->C,1,

91,G->T,1,

173,T->C,1,

180,T->C,1,

358,C->T,1,

5,T->A,1,

331,T->C,1,

93,T->C,1,

19,A->C,1,

331,T->C,1,

139,T->C,1,

178,G->A,1,

331,T->C,1,

70,T->A,1,

382,T->A,1,

368,C->T,1,

5,T->A,1,

37,C->A,1,

220,A->T,1,

225,A->G,1,

207,T->C,1,

136,G->A,1,

166,T->C,1,

166,T->C,1,

318,G->A,1,

195,T->C,1,

312,T->C,1,

195,T->A,1,

279,C->T,1,

215,T->C,1,

312,T->C,1,

271,T->C,1,

347,T->C,1,

312,T->C,1,

166,T->C,1,

Sequence: lacZ

CTGGTGAAAAGAAAAACCACCCTGGCGCCCAATACGCAAACCGCCTCTCCCCGCGCGTTG

GCCGATTCATTAATGCAGCTGGCACGACAGGTTTCCCGACTGGAAAGCGGGCAGTGAGCG

CAACGCAATTAATGTGAGTTAGCTCACTCATTAGGCACCCCAGGCTTTACACTTTATGCT

TCCGGCTCGTATGTTGTGTGGAATTGTGAGCGGATAACAATTTCACACAGGAAACAGCTA

TGACCATGATTACGAATTCACTGGCCGTCGTTTTACAACGTCGTGACTGGGAAACCCTGG

CGTTACCCAACTTAATCGCCTTGCAGCACATCCCCCTTTCGCCAGCTGGCGTAATAGCGA

AGAGGCCCGCACCGATCGCCCTTCCCAACAGCTGCGCAGC

**Supplementary Figure S4. Statistical analysis of mutable motifs in sites of somatic mutations and surrounding regions. This is a simplified example. The excess of mutations in of CpG motif was calculated using the ratio Fm/Fn, where Fm is the mean weight of mutable motifs in the positions of each somatic mutation (in C/G or A/T positions), and Fn the mean weight of mutable motifs in C/G or A/T positions without mutations in the DNA neighborhood (all un-mutated positions in the 120 bp window).**

**Position -1 0 Position 0 (position of mutation)**

**A 0 0 is not used in calculations**

**Weight matrix: T 0 0**

**G 0 1**

**C 1 0**

**CpG weight = 1, non-CpG weight = 0**

**----atCtGCGaaC G CttCGtGtta-----**

**----tttCGaCCtt C CttCCCtaaa-----**

**----CGCGttatta C GtaaatttCC-----**

**Neighborhood Neighborhood**

**^**

**Position of mutation**

**C/G shown in capital letters**

**Positions of mutation:**

**2 mutations in CpG, 1 mutation in non-CpG**

**Mean weight Fm = 2 /(2+1)= 0.66**

**Neighborhood (surrounding regions):**

**12 C:G nucleotides belongs to CpG, 14 C:G positions in non-CpG context**

**Mean weight Fn = 12 /(12+14)= 0.46**

**Supplementary Figure S5. Correlation of pol η (eta) and θ (theta) mutable motifs and the sequence context of somatic mutations. For the actual data, see Supplementary Tables S1 and S2. Intensities of gray color correspond to** **Ratio values (the Ratio being the mean weight of mutated sites divided by the mean weight of non-mutated sites). The unweighted pair group method, with arithmetic mean (UPGMA) clustering of ratio values for the pol η and θ footprints and tissues, is shown to the left and top. The upper left panel shows the distribution of the studied Ratio values and correspondence of the Ratio values and color intensity (the darker colors correspond to the higher correlation values).**

**
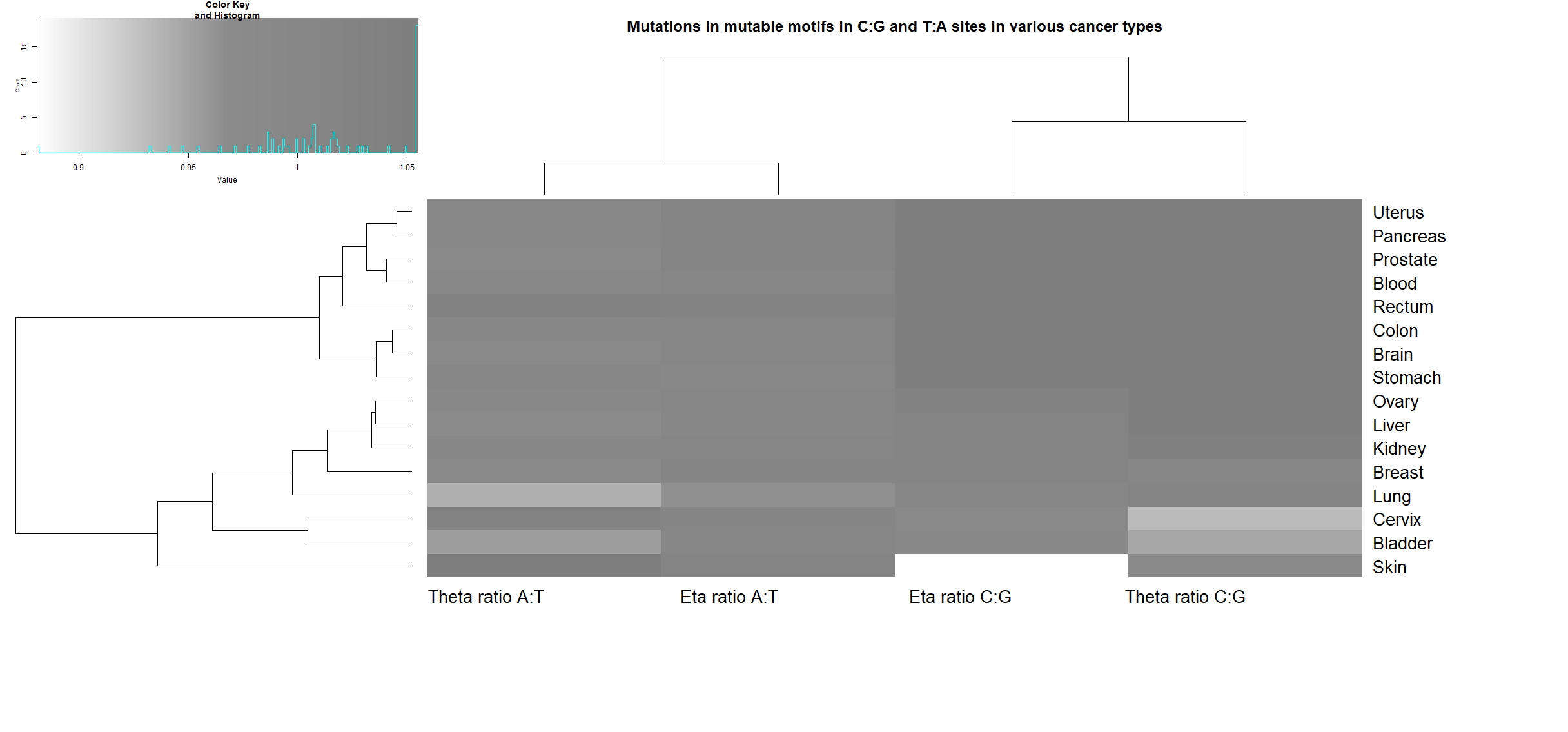
**

**Supplementary Figure S6.** **Heatmap of Pearson’s pairwise correlation coefficient for methylation profiles in driver genes. The darker colors correspond to the higher correlation values****, scale from 0.9 to 1. Statistical analysis of CC values (multiplied by 100, this is a rough estimate) using the χ^2^ test suggested that the observed distributions are not different from uniform distributions (P = 0.39 for driver genes). Patient order: 1 - DO27861, 2 -DO27863, 3 - DO27865, 4 - DO27819, 5 - DO27815, 6 - DO27821, 7 - DO27823, 8 -DO27825, 9 - DO27809, 10 - DO27801, 11 - DO27803, 12 - DO27805, 13 - DO27795, 14 - DO27797, 15 -DO27799, 16 - DO27773, 17 - DO27775, 18 - DO27781, 19 - DO27785, 20 - DO27787, 21 - DO27764, 22 - DO27763, 23 - DO27765, 24 - DO27767, 25 - DO27769, 26 -DO22111.**

**

**

**Supplementary Figure S7. Heatmap of Pearson’s pairwise correlation coefficient for methylation profiles in non-driver genes. The darker colors correspond to the higher correlation values, scale from 0.9 to 1. Statistical analysis of CC values (multiplied by 100, this is a rough estimate) using the χ^2^ test suggested that the observed distributions are not different from uniform distributions (P = 0.47 for non-driver genes). Patient order: 1 - DO27861, 2 - DO27863, 3 - DO27865, 4 - DO27819, 5 - DO27815, 6 - DO27821, 7 - DO27823, 8 -DO27825, 9 - DO27809, 10 - DO27801, 11 - DO27803, 12 - DO27805, 13 - DO27795, 14 - DO27797, 15 -DO27799, 16 - DO27773, 17 - DO27775, 18 - DO27781, 19 - DO27785, 20 - DO27787, 21 - DO27764, 22 - DO27763, 23 - DO27765, 24 - DO27767, 25 - DO27769, 26 -DO22111.**

**

**

**Supplementary Figure S8. Schematic representation of the procedure used for construction of Table 4. Only CpG sites that overlap with somatic mutations are shown (the number of circles is smaller than in the Figure 1). Each circle represents a methylated CpG site, with its size reflecting the methylation level. The set “1” (the methylation levels are larger than 75%) is compared to set “2” (the methylation levels are smaller than 75%). Red “X” stands for CpG sites that overlap with mutable motifs and somatic mutations.**


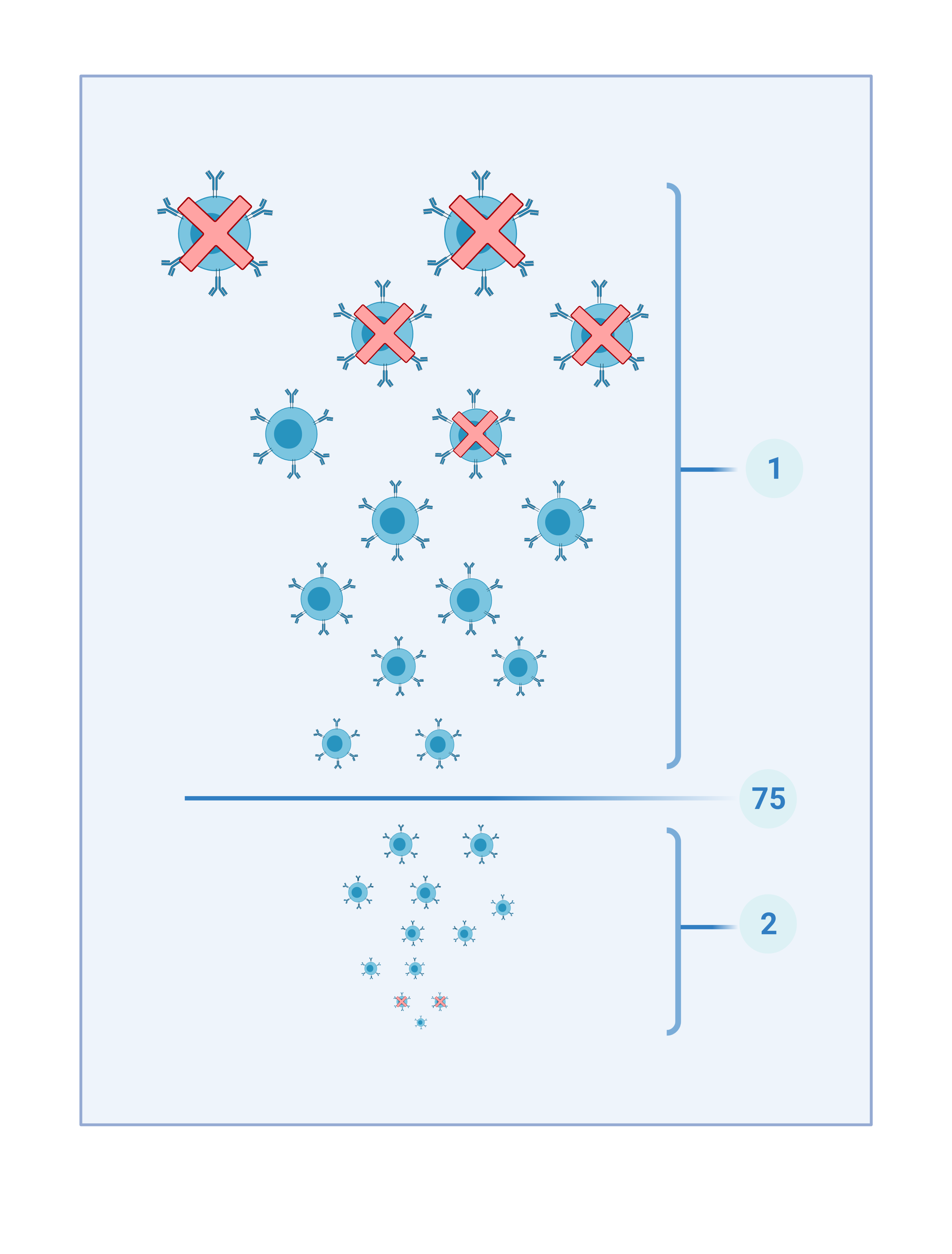

Supplement: Supplementary file 1 [file Data_Sheet_1.doc]
